# Role variability of surface chemistry and surface topography in anti-icing performance

**DOI:** 10.1016/j.isci.2024.111039

**Published:** 2024-09-28

**Authors:** Wei Weng, Mizuki Tenjimbayashi, Masanobu Naito

**Affiliations:** 1 Research Center for Macromolecules and Biomaterials, National Institute for Materials Science (NIMS), 1-2-1 Sengen, Tsukuba, Ibaraki 305-0047, Japan; 2 Research Center for Materials Nanoarchitectonics (MANA), National Institute for Materials Science (NIMS), 1-1 Namiki, Tsukuba, Ibaraki 305-0044, Japan

**Keywords:** Chemistry, Surface chemistry, Material science

## Abstract

Largely varied anti-icing performance among superhydrophobic surfaces remains perplexing and challenging. Herein, the issue is elucidated by exploring the roles of surface chemistry and surface topography in anti-icing. Three superhydrophobic surfaces, i.e., gecko-like, petal-like, and lotus-like surfaces, together with smooth hydrophobic and hydrophilic surfaces, are prepared and compared in ice nucleation temperature under both non-condensation and condensation conditions. As a result, in non-condensation condition, water droplet freezing is caused by interfacial heterogeneous nucleation, wherein both surface chemistry and surface topography contribute to deferring freezing, and the former is dominant. In condensation condition, the freezing strongly correlates to condensation frosting. Surface chemistry maintains as a strong deterrent, whereas surface topography has two competing effects on the freezing. The paper deepens the understanding of water freezing on superhydrophobic surfaces, unravels the correlation between superhydrophobicity and anti-icing, and provides design guidelines on application-oriented anti-icing surfaces.

## Highlights

• Freezing mechanisms of water droplets changing with environment are revealed
• Roles of surface chemistry and surface topography in anti-icing are analyzed
• Correlation between superhydrophobicity and anti-icing is elucidated

## Graphical abstract

**Figure:**
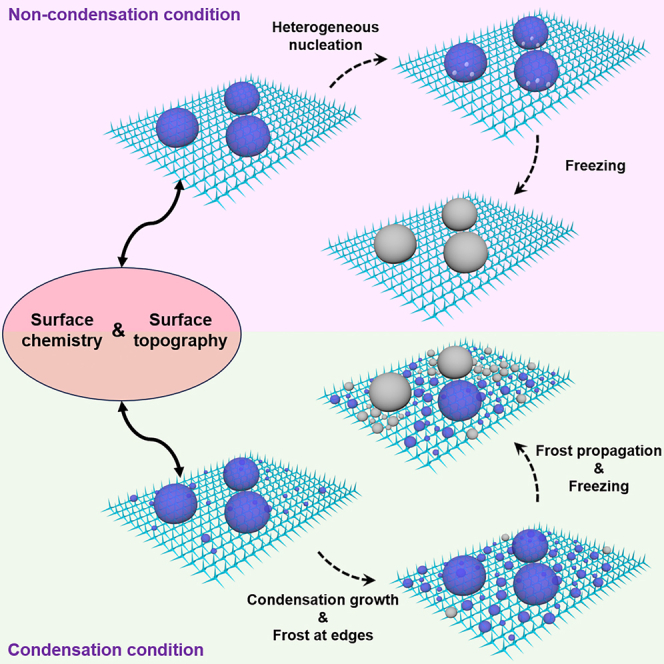

## Introduction

Icing, a ubiquitous phenomenon, however brings about devastating disasters to air and road traffic, malfunction of solar cells and wind turbines, and plummeted crop production.^1^ To this end, much effort has been devoted to anti-icing, among which superhydrophobic strategy is a widely adopted recipe to lower ice nucleation temperature (INT) and prolong freezing delay time (FDT) in undercooling conditions.^2^^,^^3^^,^^4^ Although the anti-icing performance of superhydrophobic surfaces has been intensively studied over the last decade, it is still in dispute, which can be reflected from a wide INT gap of over 10°C and two orders of magnitude deviation for FDT.^5^^,^^6^^,^^7^^,^^8^^,^^9^^,^^10^^,^^11^^,^^12^

Apparently, the varied anti-icing performance was derived from different superhydrophobic genres. Superhydrophobic surfaces are realized via regulating both surface chemistry and surface topography.^13^^,^^14^ Employment of low-surface-energy materials or coatings is necessary for superhydrophobicity. Designs of surface topographies owning multi-scale hierarchical roughness and trapped air pockets are also indispensable. Since the revelation of dual-scale nano-/micro-textures for lotus leaves, a plethora of biomimetic superhydrophobic surfaces have sprouted up, possessing distinctive topographies, e.g., lotus-like, petal-like, and gecko-like.^15^^,^^16^^,^^17^^,^^18^^,^^19^ Generally, such characters of superhydrophobic surfaces benefit the anti-icing performance by lifting the energy barrier for ice nucleation and/or lowering the heat transfer between surfaces and water droplets.^20^^,^^21^^,^^22^^,^^23^^,^^24^

Moreover, the varied anti-icing performance of superhydrophobic surfaces was tested in different conditions. The effects of droplet size and cooling rate can be well explained by classical nucleation theory (CNT).^25^ In addition, relative humidity (RH) has a profound influence on the freezing of water droplets. On one hand, high RH probably destabilizes the trapped air pockets and increases surface-droplet contact in a direct manner.^26^^,^^27^^,^^28^ On the other hand, the ambient water vapor may condense on surfaces to interfere the freezing of water droplets in an indirect manner.^29^^,^^30^ Therefore, to find out the origin of anti-icing performance variation among superhydrophobic surfaces, the roles of surface chemistry and surface topography in anti-icing under different conditions should be traced and explicated, which though is absent to the best of our knowledge.

Herein, five samples, i.e., smooth hydrophilic surface, smooth hydrophobic surface, gecko-like, petal-like, and lotus-like superhydrophobic surfaces, are prepared. And their anti-icing properties are compared in two test conditions, which are condensation and non-condensation conditions. Briefly, the respective INT of gecko-like, petal-like, and lotus-like superhydrophobic surfaces is −30.5, −30.5, and −30.4°C in non-condensation condition, which goes to −15.0, −12.8, and −16.1°C in condensation condition accordingly. The nearly same INT values in non-condensation condition deviate from each other in condensation condition, and the INT values in non-condensation condition are far superior to the counterparts in condensation condition. The reason is that the freezing mechanism of water droplets experiences a change when condensation occurs, and thus the roles of surface chemistry and surface topography vary, resulting in such distinct anti-icing performance.

## Results

### Design and characterization of superhydrophobic surfaces

In this work, polydimethylsiloxane (PDMS) as low-surface-energy material, together with gecko-like, petal-like, and lotus-like topographies, was applied to construct three superhydrophobic surfaces. To be mentioned, gecko-like surface is nano-textured, and petal-like and lotus-like surfaces are nano-/micro-textured with different extents of roughness.^15^^,^^31^ Three superhydrophobic surfaces, which were either spin-coated or dip-coated on glass slides, are all composed of micrometer-sized ZnO tetrapods (Figure S1) and PDMS, where ZnO tetrapods are wrapped and glued by PDMS. Additionally, two control samples are glass slides as smooth hydrophilic surface and PDMS-coated glass slides as smooth hydrophobic surface.

**Figure 1:**
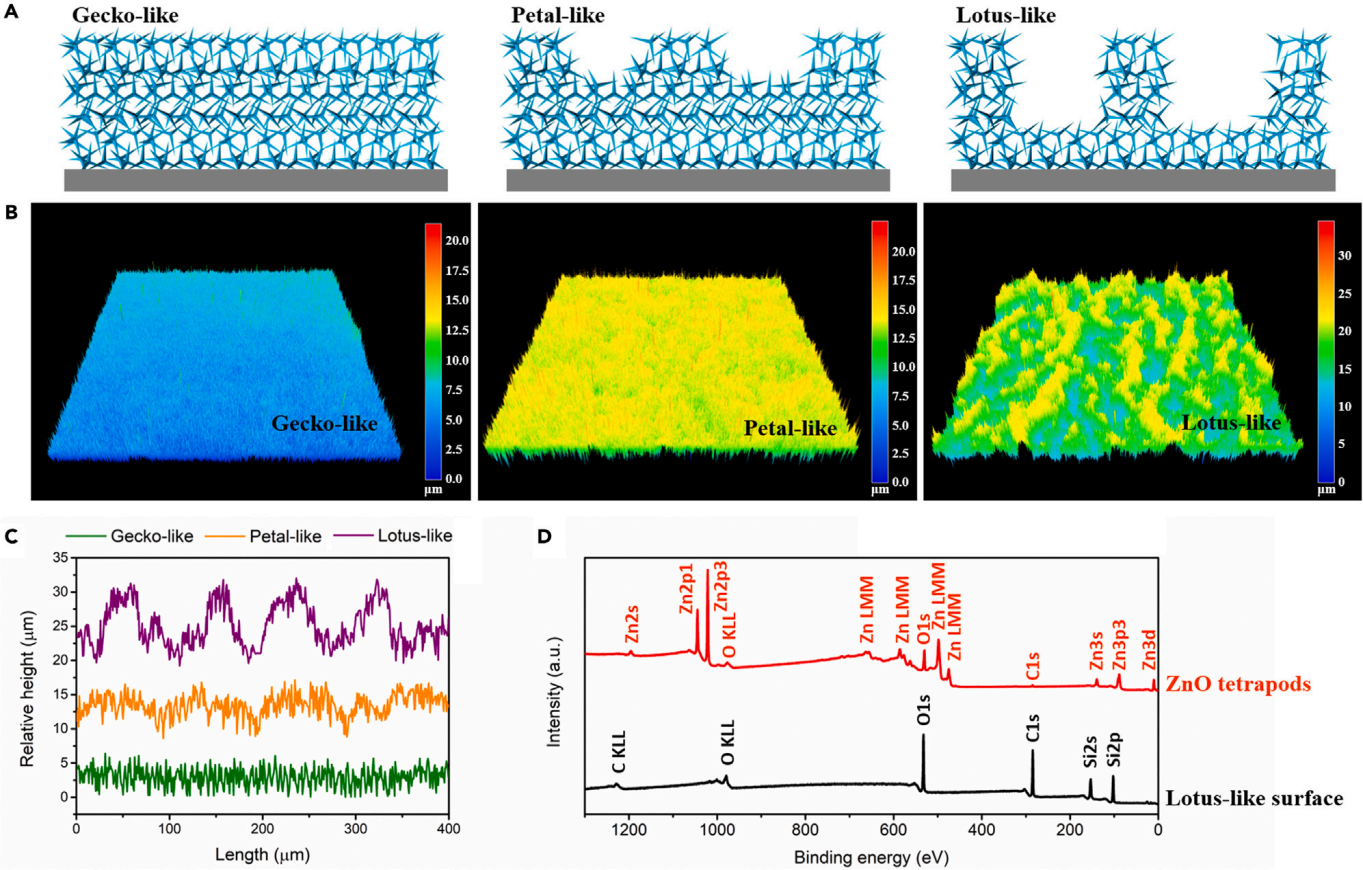
3D morphology and elemental characterization (A) Schematics of surface topographies for gecko-like, petal-like, and lotus-like surfaces. (B) 3D images with areas of 750 μm × 750 μm for gecko-like, petal-like, and lotus-like surfaces by laser scanning confocal microscopy. (C) Surface height profiles of gecko-like, petal-like, and lotus-like surfaces. (D) XPS survey spectra of ZnO tetrapods and lotus-like surface composed of ZnO tetrapods and PDMS.

Schematics of three superhydrophobic surfaces are illustrated in Figure 1A, in which light blue tetrapods refer to PDMS-wrapped ZnO. With a fine control of the tetrapods’ distribution, three different topographies using same materials have been achieved. Observed from laser scanning confocal microscope, three-dimensional (3D) images of superhydrophobic surfaces are presented in Figure 1B, whose height profiles are shown in Figure 1C. The morphology of gecko-like surface looks like vertical needle arrays, and its roughness (arithmetical mean height, *Ra*) is 0.73 μm. Micrometer-sized protrusions are generated in petal-like and lotus-like surfaces, resulting in mountain-valley morphology. And the valleys for lotus-like surface are deeper and wider than those in petal-like surface, which brings about larger roughness to lotus-like surface (*Ra* = 2.47 μm) than petal-like surface (*Ra* = 1.12 μm). Surface chemical composition was probed by X-ray photoelectron spectroscopy (XPS) for both superhydrophobic surfaces and ZnO powders. The diameter of test areas was 200 μm. No Zn element was detected for lotus-like surface (Figure 1D), which demonstrates that ZnO tetrapods are fully covered by PDMS. Thus, the surface chemistry of superhydrophobic surfaces and PDMS is same as we anticipated.

**Figure 2:**
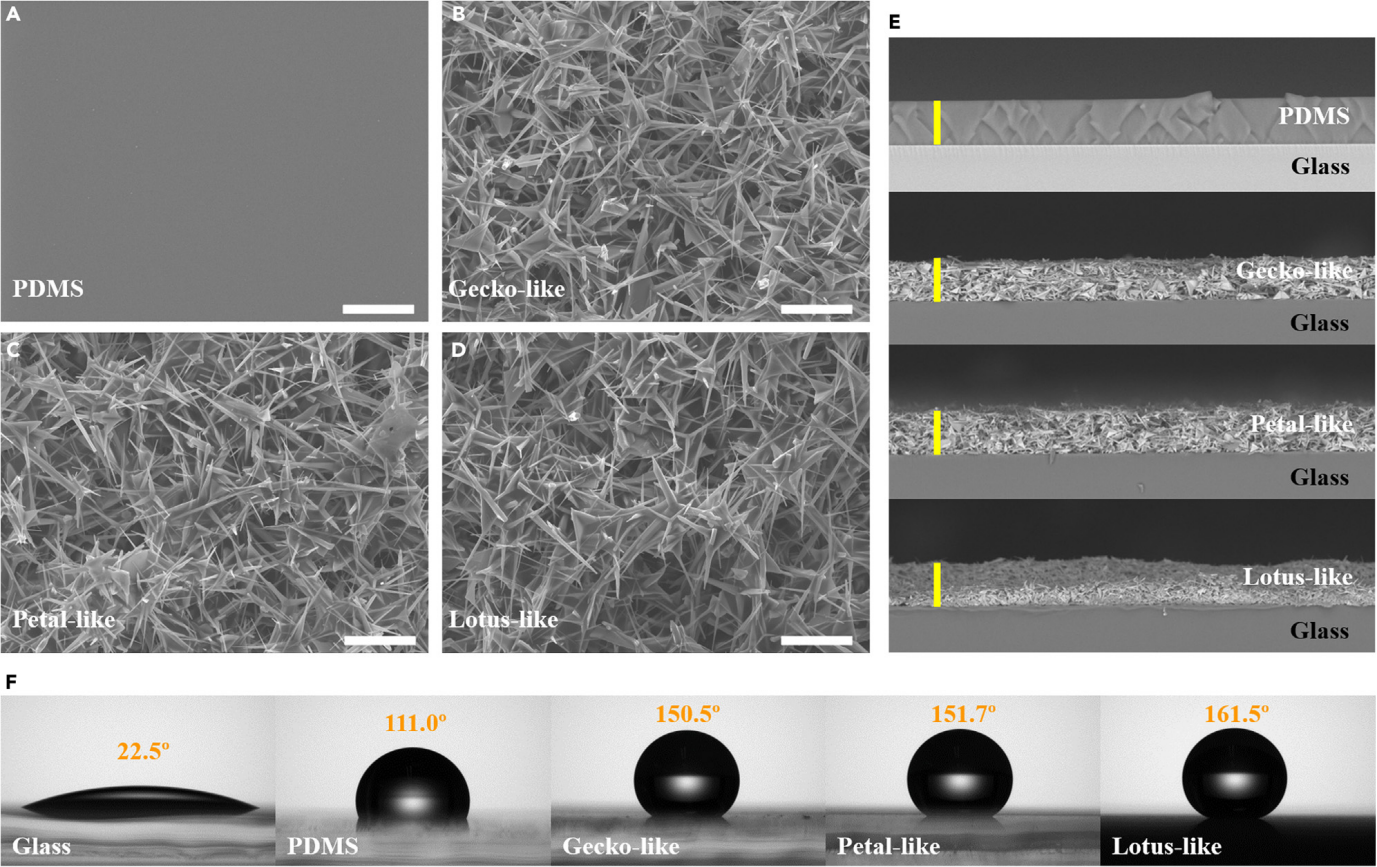
SEM morphology and wettability characterization (A–D) Top-view SEM images of (A) PDMS, (B) gecko-like, (C) petal-like, and (D) lotus-like surfaces. White scale bars are 10 μm. (E) Cross-sectional SEM images of PDMS, gecko-like, petal-like, and lotus-like surfaces. Yellow scale bars are 20 μm. (F) CA test photos of water droplets of 8 μL on glass, PDMS, gecko-like, petal-like, and lotus-like surfaces. See also Figures S1 and S2.

Moreover, surfaces were characterized by scanning electron microscopy (SEM). PDMS surface is flat (Figure 2A). On the opposite, superhydrophobic surfaces are rough (Figures 2B–2D). It seems there are no big differences among these high-magnification images, which means that the nano-textures for three superhydrophobic surfaces are similar. Their morphology discrepancy lies in their micro-textures, which is manifest in Figures 1B and 1C. To be mentioned, PDMS surface and superhydrophobic surfaces possess same thicknesses of 20 μm (Figure 2E), excluding the effect of thickness on the anti-icing performance. Furthermore, static contact angles (CAs) for glass, PDMS, gecko-like, petal-like, and lotus-like surfaces are 22.5 ± 0.5°, 111.0 ± 0.5°, 150.5 ± 0.6°, 151.7 ± 1.1°, and 161.5 ± 1.3°, respectively (Figures 2F and S2A). Advancing CAs (*θ*_*adv*_) and receding CAs (*θ*_*rec*_) can be found in Figure S2B. Lotus-like surface exhibits small CA hysteresis (CAH, the difference between *θ*_*adv*_ and *θ*_*rec*_) of 8°, and gecko-like and petal-like surfaces own large CAH around 30°. As a result, gecko-like, petal-like, and lotus-like superhydrophobic surfaces are confirmed by morphology and wettability results.^31^^,^^32^

### Ice nucleation in non-condensation condition

INT of water droplets on different surfaces was first tested in an environmental chamber, where surfaces’ temperature was same to the chamber temperature (Figure S3A). To be emphasized, water droplets were cooled slowly and simultaneously with surfaces and the environment. Thereby, heat transfer between droplets and surfaces was negligible.^33^ Moreover, when the chamber temperature was decreased from 20°C to −35°C at 1.0°C min^−1^ (cooling rate for INT test), the chamber RH changed accordingly and followed a route (Figure S4A). Thus, the chamber temperature was always higher than the corresponding dew point (Figure S4B), which guaranteed a non-condensation test condition.

**Figure 3:**
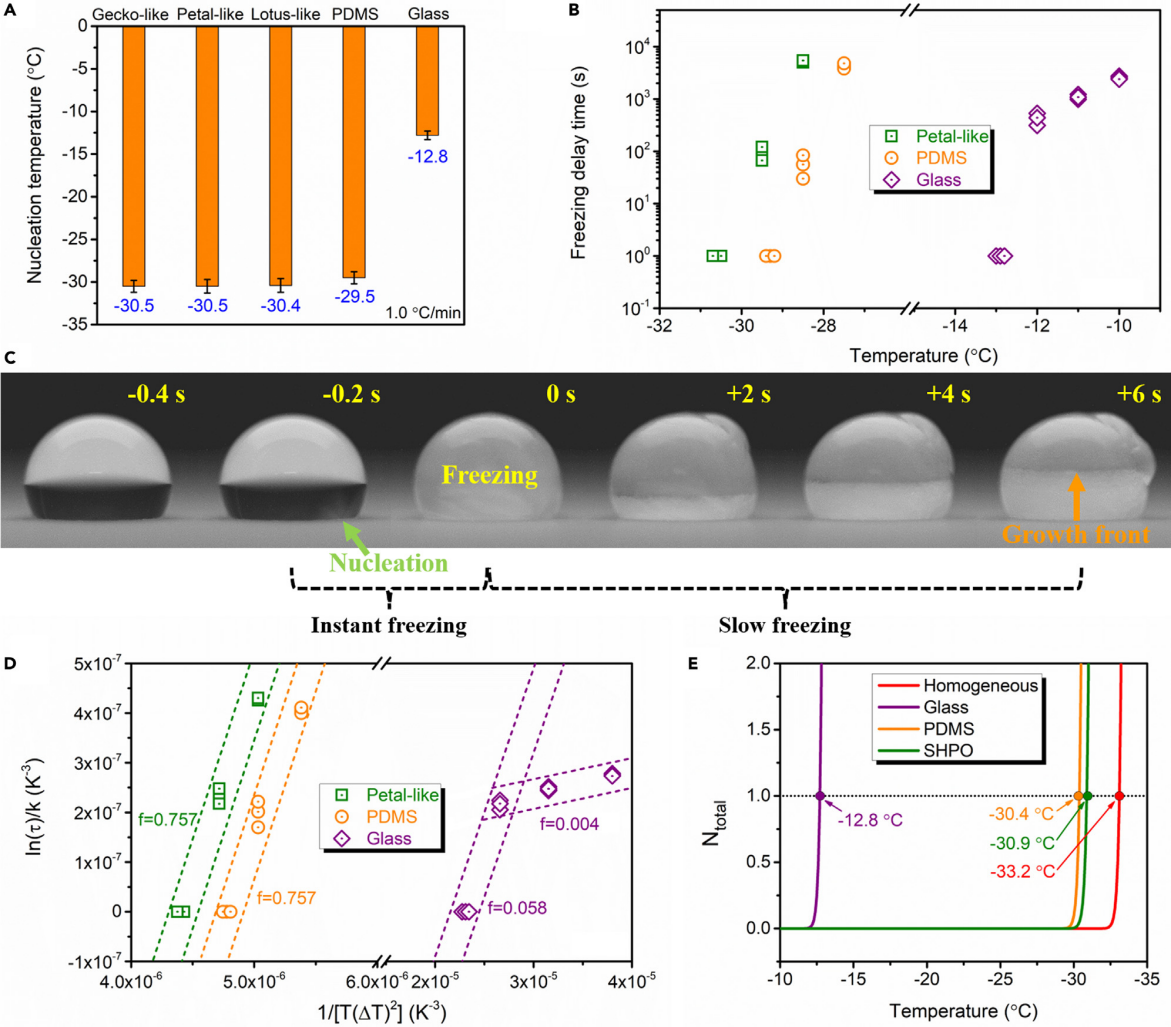
Anti-icing performance in non-condensation condition (A) INT of water droplets on glass, PDMS, gecko-like, petal-like, and lotus-like surfaces. Data are represented as mean ± SD. (B) FDT of water droplets on glass, PDMS, and petal-like surfaces. (C) Snapshots of the freezing process of a water droplet (10 μL) on lotus-like surface during INT test. (D) Experimental dots and linear fitting of ln(*τ*)/*k* versus 1/[*T*(*ΔT*)^2^] for water droplets on glass, PDMS, and petal-like surfaces. (E) CNT-based theoretical INT of water droplets via homogeneous nucleation and heterogeneous nucleation on glass, PDMS, and superhydrophobic (SHPO) surfaces. See also Figures S5–S12.

As seen from Figure 3A, the INT for glass, PDMS, gecko-like, petal-like, and lotus-like surfaces is −12.8, −29.5, −30.5, −30.5, and −30.4°C, respectively. Obviously, surface chemistry has a significant effect on INT. Glass surface is hydrophilic and presents the highest INT, whereas PDMS surface is hydrophobic, which shows far lower INT. The lower INT of superhydrophobic surfaces than PDMS surface should be ascribed to the introduction of surface textures since they have close surface chemistry. However, three superhydrophobic surfaces exhibit nearly same INT regardless of their varied topographies. FDT data are shown in Figure 3B. Similarly, water droplets on superhydrophobic surfaces have the strongest resistance against freezing, and those on glass surface are the earliest to freeze. In detail, the FDT for glass surface at −10°C is about 2,500 s. For PDMS surface, the FDT at −27.5°C is more than 4,000 s. As to petal-like surface, it is more than 5,000 s at −28.5°C.

Figure 3C illustrates the freezing process of a water droplet on lotus-like surface, which was monitored by a high-speed camera. The whole process that is typical for supercooled droplets can be divided into two stages: stage-one instant freezing that completes in less than 1 s and stage-two slow freezing.^29^ The first stage was able to be identified by the brightness change, which was so obvious that bare eyes could notice. Therefore, the temperature upon stage-one freezing was taken as INT. Also, from Figure 3C, it was found that the nucleation was initiated at the surface-droplet interface and the crystallization front moved toward the top, verifying heterogeneous nucleation.^34^ Actually, homogeneous nucleation near the droplet-air interface was never observed in over 100 nucleation tests because it is prone to occur at low humidity (e.g., 30% RH).^34^

To clarify the contributions of surface chemistry and surface topography, CNT was applied. The role of surface chemistry can be interpreted by the heterogeneous nucleation parameter *f*, which has a direct relationship with FDT:^1^^,^^35^

(Equation 1) $$\ln\left(\tau \right)\propto f\frac{\Delta G}{k_{B}T}=f\frac{k}{T{\left(\Delta T\right)}^{2}}\text{,}$$

(Equation 2) $$k=\frac{16\pi }{3}\frac{v_{ice}^{2}\sigma_{iw}^{3}}{{\left(\Delta S_{m}\right)}^{2}k_{B}}\text{,}$$

where *τ* is the FDT; *ΔG* is the Gibbs-free energy for the formation of a critical ice embryo; *k*_*B*_ is the Boltzmann constant; *T* is the temperature; *f*, in a range of 0–1, is the interfacial correlation factor denoting the reduction of the Gibbs energy barrier due to the presence of heterogeneous nuclei; *ΔT* = *T*_*m*_ – *T* (*T*_*m*_ is the ice melting temperature); *v*_*ice*_ is the volume of a water molecule in ice; *σ*_*iw*_ is the interfacial tension between ice and water; and *ΔS*_*m*_ denotes the melting entropy per molecule.^35^^,^^36^^,^^37^

Actually, *f* is a function of chemical affinity (denoted as *m*) and structural compatibility (denoted as *R′*) between the ice embryo and surfaces.^1^ *m* ≈ cos*θ*, in which *θ* is the CA of the ice embryo on surfaces. *R'* = *R*/*r*_*c*_, where *R* is the radius of curvature of surface textures and *r*_*c*_ is the radius of the critical ice embryo. Notably, the structural part of *f* does not work unless *R'* < 10.^1^ *r*_*c*_ has the form of^1^

(Equation 3) $$r_{c}=\frac{2v_{ice}\sigma_{iw}}{\Delta S_{m}\left(T_{m}-T\right)}\text{.}$$

*v*_*ice*_ has the form as below:^36^

(Equation 4) $$v_{ice}=\frac{M_{w}}{N_{a}\rho_{0}}{\left(1-0.05294T_{r}-0.05637T_{r}^{2}-0.002913T_{r}^{3}\right)}^{-1}\text{,}$$

where *M*_*w*_ is the molar mass of water, *N*_*a*_ is the Avogadro constant, *ρ*_*0*_ is the density of ice at the ice melting point (*T*_*m*_), and *T*_*r*_ = (*T* – *T*_*m*_)/*T*_*m*_.

*σ*_*iw*_ can be expressed as^36^

(Equation 5) $$\sigma_{iw}=0.03298+0.012048T_{r}-0.46705T_{r}^{2}\text{.}$$

Regarding *ΔS*_*m*_, it can be estimated as *L*_*m*_/*T*_*m*_, where *L*_*m*_ is the latent heat of ice melting.^35^ Subsequently, *r*_*c*_ as a function of temperature was calculated (Figure S5). It is 1.56 nm at −30°C, which is consistent with other reports.^38^^,^^39^^,^^40^ On the other hand, *R* should be larger than tens of nanometers because of the micrometer-sized ZnO tetrapods. Thereby, *f* is only relevant to surface chemistry with the following equation:^1^

(Equation 6) $$f=\frac{1}{4}\left(2-3m+m^{3}\right)\text{.}$$

Prior to calculating *f*, *k* was computed (Figure S6). Afterward, using the FDT data in Figure 3B, plots of ln(*τ*)/*k* versus 1/[*T*(*ΔT*)^2^] were drawn in Figure 3D for glass, PDMS, and petal-like surfaces. *f* was then directly obtained from the slopes of lines. *f* values for both PDMS and petal-like surfaces approximate 0.757, demonstrating their same surface chemistry in accordance with the XPS result. On the contrary, glass surface presents two-stage *f* values: one is about 0.058 near the INT, and the other is around 0.004 when the temperature is away from the INT. Moreover, from *f* of 0.757, *θ* of 111° is got using Equation 6, which coincides with the CA of PDMS surface. With respect to *f* of 0.058, *θ* equals 45°, which was suggested to be the boundary between hydrophilicity and hydrophobicity.^25^ Hence, the role of surface chemistry of superhydrophobic surfaces is determined by the wettability of a smooth hydrophobic counterpart.

Furthermore, the role of surface topography can be discerned from the integration of heterogeneous nucleation rate *J*_*het*_ using the Poisson process to predict INT^25^^,^^35^:

(Equation 7) $$\int_{T_{m}}^{T_{f}}J_{het}SdT/C=N_{total,}$$

(Equation 8) $$J_{het}=N_{s}\frac{k_{B}T}{h}\exp\left(-\frac{\Delta Q_{diff}}{k_{B}T}\right)\exp\left(-\frac{f\Delta G}{k_{B}T}\right)\text{,}$$

where *T*_*f*_ is the undercooling temperature, *C* is the cooling rate, *S* is the area of surface on which heterogeneous nucleation may happen, *N*_*total*_ is the total number of nucleation sites, *N*_*s*_ is the surface-based number density of water molecules in the liquid parent phase, *h* is the Planck constant, and *ΔQ*_*diff*_ is the activation energy for the transfer of a water molecule across the water-ice boundary.^35^ The effect of surface topography is related to *S*. On one hand, *S* tends to shrink with enlarging CA. As water droplets can be deemed as a spherical cap,^41^ *S* only depends on CA when the volume of water droplets (*V*) is fixed as shown in the following equation:

(Equation 9) $$S=\pi {\left(\sqrt[{3}]{\frac{3V}{\pi \left(2+\cos\;\theta \right){\left(1-\cos\;\theta \right)}^{2}}}\sin\;\theta \right)}^{2}$$

CAs of PDMS, gecko-like, petal-like, and lotus-like surfaces are 111.0°, 150.5°, 151.7°, and 161.5°, respectively. Accordingly, the *S* for PDMS surface is 4.3, 4.6, and 10.4 times the values for gecko-like, petal-like, and lotus-like surfaces, respectively (Figure S7).

On the other hand, *S* diminishes due to the trapped air pockets when textured surfaces are applied.^31^^,^^42^ The areal fraction of air pockets (*ϕ*) for gecko-like surface can be calculated using Cassie-Baxter equation:

(Equation 10) cos(150.5°)=ϕcos(180°)+(1−ϕ)cos(111°),

from which *ϕ* equals 79.8%. Hence, the decrease factor is 4.95. For petal-like surface, it should be discussed in two aspects: first, *S* is increased due to the water impalement into its micro-textures,^31^ which is indicated by a factor of 2.49/2.43 that are the roughness factors (ratio of total surface area to projected area) of petal-like and gecko-like surfaces, respectively. Second, *S* is decreased due to the trapped air pockets in its nano-textures by a factor of 4.95 since petal-like and gecko-like surfaces possess similar nano-textures. As to lotus-like surface, its micro-textures are free of water impalement, whose areal fraction (*ψ*) can be calculated using Cassie-Baxter equation:

(Equation 11) cos(161.5°)=ψcos(180°)+(1−ψ)cos(150.5°),

from which *ψ* is 60.1%. Therefore, considering the trapped air pockets in both nano- and micro-textures, the decrease factor is 12.4. In sum, the *S* for PDMS surface is 21.3, 22.2, and 129.0 times the values for gecko-like, petal-like, and lotus-like surfaces, respectively.

However, the situations below 0°C are different from the one at room temperature. CAs with decreasing temperature were measured (Figure S8). A moment before icing, the CAs of PDMS, gecko-like, petal-like, and lotus-like surfaces are 86.8°, 135.1°, 133.1°, and 133.8°, respectively. All surfaces suffered a large CA drop, which is mainly ascribed to being in receding state and evaporation-driven water impalement into textures.^43^ Nevertheless, the air pockets in nano-textures should be maintained.^44^^,^^45^ For clarity, schematics of water droplets on three superhydrophobic surfaces at room and sub-zero temperatures are presented (Figure S9). Besides the CA change for all surfaces, the micro-textures of lotus-like surface free of water impalement at room temperature is occupied by water before icing, which was further proved by the 3D distribution of fluorescent dye residue (Figure S10). Water droplets doped with a slight amount of Rhodamine B were placed on surfaces either at room temperature for 20 min or being cooled to −20°C at a rate of 1°C min^−1^ before removal. The fluorescence intensity corresponding to the cooling situation is much stronger than that at room temperature, revealing water penetration into the micro-textures of lotus-like surface. On the contrary, the fluorescence intensities in two situations are similar for gecko-like (Figure S11) and petal-like (Figure S12) surfaces. Consequently, *S*_*PDMS*_ = (3.2 × $\frac{1}{1-\phi }$) *S*_*gecko-like*_ = 15.8 × *S*_*gecko-like*_. *S*_*PDMS*_ = (3.0 × $\frac{1}{1-\phi }$ × $\frac{2.43}{2.49}$) *S*_*petal-like*_ = 14.5 × *S*_*petal-like*_, and *S*_*PDMS*_ = (3.1 × $\frac{1}{1-\phi }$ × $\frac{2.43}{2.72}$) *S*_*lotus-like*_ = 13.7 × *S*_*lotus-like*_, where 2.72 is the roughness factor of lotus-like surface. Hence, the comparable *S* in undercooling conditions together with the same surface chemistry led to the similar INT for three superhydrophobic surfaces.

Finally, theoretical INT of water droplets on glass, PDMS, and superhydrophobic surfaces was predicted using Equations 7 and 8. Here, *N*_*total*_ was assigned to ∼1 for the fitting.^25^ Water droplet of 10 μL and cooling rate of 1.0°C min^−1^ were used, which are same to the experimental setup. Also, $\Delta Q_{diff}=\frac{k_{B}T^{2}E}{{\left(T-T^{*}\right)}^{2}}$, where *E* = 892 K and *T∗* = 118 K.^35^ 10^25^ m^−2^ was assigned to *N*_*s*_ of both PDMS and superhydrophobic surfaces. And 2.5 × 10^12^ m^−2^ was chosen for *N*_*s*_ of glass surface since strong binding affinity between water and hydrophilic surfaces prevents the first water layer from being arranged into the ice structure.^1^^,^^37^^,^^46^ As a result, the theoretical INT of glass, PDMS, and superhydrophobic surfaces reaches −12.8, −30.4, and −30.9°C, respectively. Additionally, homogeneous INT was also forecasted using the following equations:^35^

(Equation 12) $$\int_{T_{m}}^{T_{f}}J_{homo}VdT/C=N_{total,}$$

(Equation 13) $$J_{homo}=N_{v}\frac{k_{B}T}{h}\exp\left(-\frac{\Delta Q_{diff}}{k_{B}T}\right)\exp\left(-\frac{\Delta G}{k_{B}T}\right)\text{,}$$

where *V* is the bulk volume of water, and *N*_*v*_ (=3.1 × 10^28^ m^−3^) is the volume-based number density of water molecules in the liquid parent phase.^35^ The calculated value is −33.2°C, which is in accordance with other reports.^25^^,^^47^ Overall, all theoretical INT data shown in Figure 3E well match the experimental results.

### Ice nucleation in condensation condition

**Figure 4:**
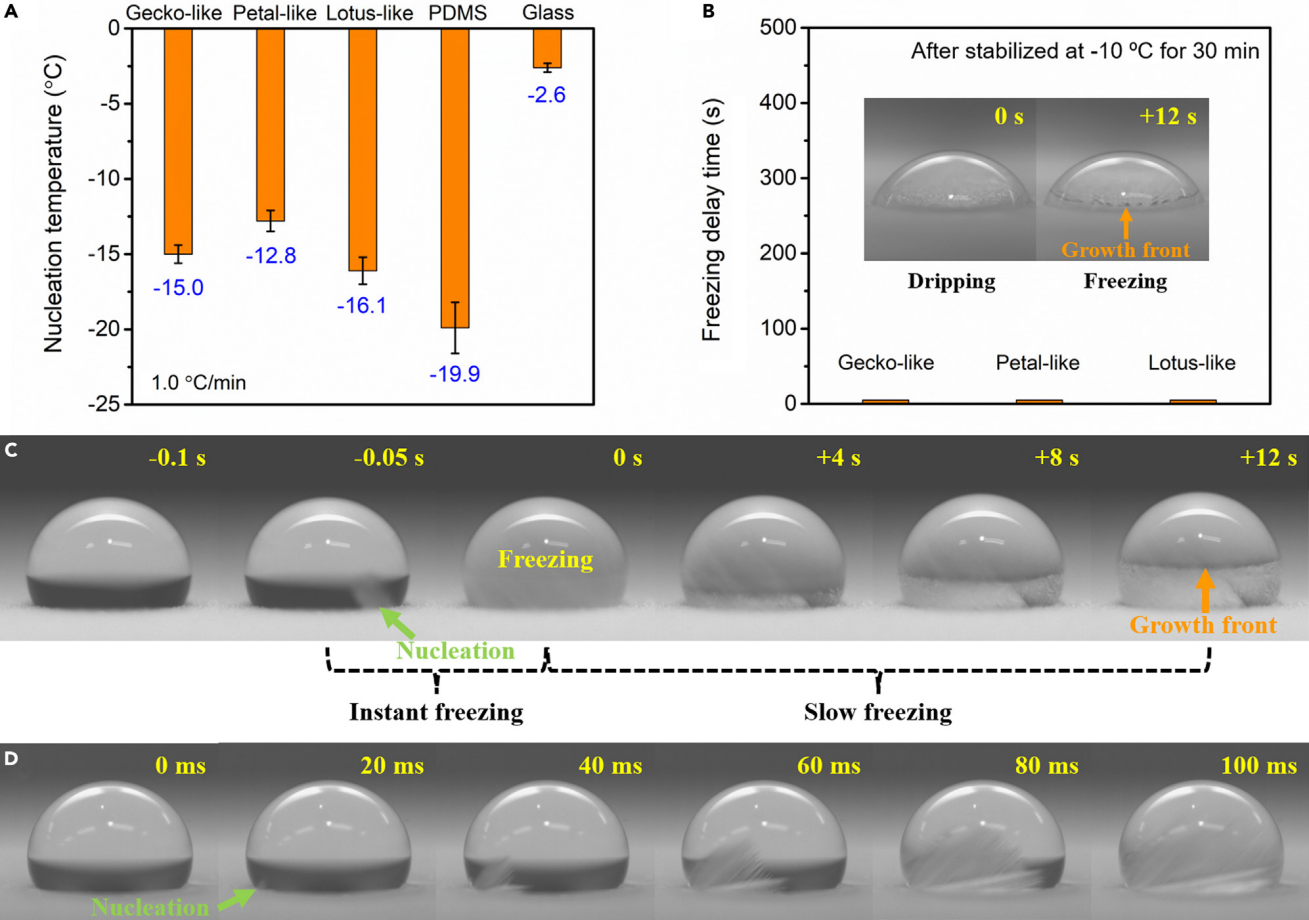
Anti-icing performance in condensation condition (A) INT of water droplets on plasma-treated glass, PDMS, gecko-like, petal-like, and lotus-like surfaces. Data are represented as mean ± SD. (B) FDT of water droplets on gecko-like, petal-like, and lotus-like surfaces. The inset shows immediate freezing of a water droplet (10 μL) once dripped on lotus-like surface during FDT test. (C) Snapshots of the freezing process of a water droplet (10 μL) on lotus-like surface during INT test. (D) High-frame-rate images of the recalescence of a water droplet (10 μL) on lotus-like surface in INT test. See also Figures S13, S14, and S16.

INT of water droplets was further tested under condensation condition in an experimental stall. Ambient temperature and RH in the stall were kept at 22 ± 1°C and 52 ± 2%, respectively, corresponding to a dew point around 11.7°C. A cooling stage was utilized, on which test surfaces were mounted (Figure S3B). Condensation would happen when surfaces’ temperature was lower than the dew point. As seen from Figure 4A, the INT for glass surface is the highest (−2.6°C) and that for PDMS surface is the lowest (−19.9°C). Gecko-like, petal-like, and lotus-like surfaces present medium INT of −15.0, −12.8, and −16.1°C, respectively. Here, the glass surface was plasma treated, which became superhydrophilic (CA ≈ 0°) as shown in Figure S13. Distinctiveness is easily found: (1) superhydrophobic surfaces possess lower INT than PDMS surface in non-condensation condition, but the opposite in condensation condition. (2) The INT for three superhydrophobic surfaces is nearly same in non-condensation condition, which deviates from each other in condensation condition. Therefore, condensation has a significant impact on the freezing of water droplets.

After superhydrophobic surfaces had been kept at −10°C for 30 min (same stabilization time in non-condensation condition), water droplets immediately froze once dripped on the surfaces, resulting in minimal FDT (Figure 4B). As seen from the inset of Figure 4B, a crystallization front was observed for a water droplet on lotus-like surface after just 12 s. More details are shown in Figure S14. It can be learnt that recalescence was absent for the freezing of water droplets during FDT test. Since condensation had lasted for a while before water droplets were dripped on surfaces, the freezing was probably induced by condensates or iced condensates.^13^^,^^48^ On the contrary, two-stage freezing still happened to the water droplets during INT test (Figure 4C). To be mentioned, no condensates were formed on surfaces before dripping of water droplets in INT test. The first freezing stage, i.e., recalescence, on lotus-like surface was clearly displayed in high-frame-rate images (Figure 4D), from which the nucleation was verified to emerge from the triple line of the droplet.

**Figure 5:**
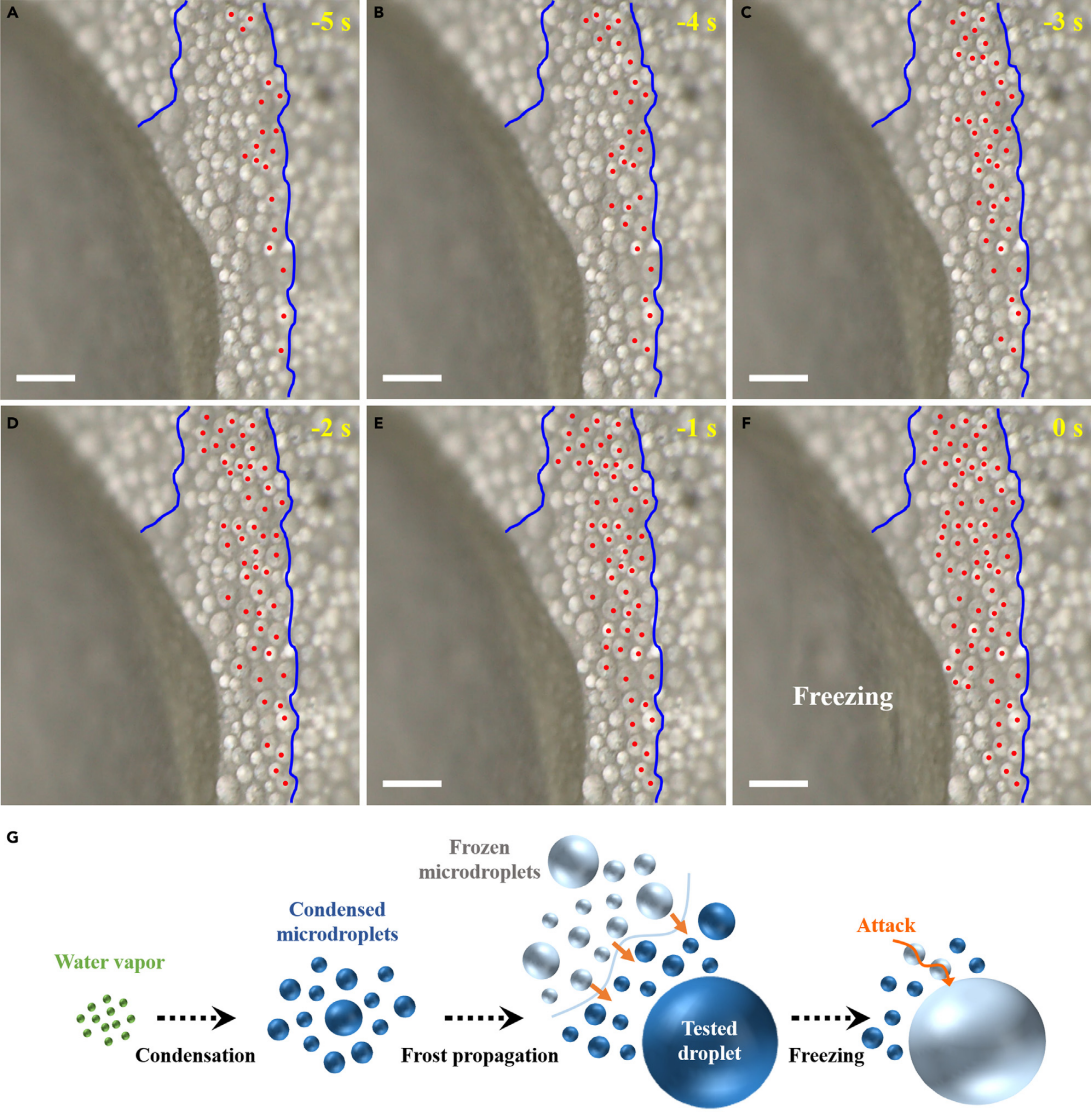
Freezing mechanism in condensation condition (A–F) Time-lapse images of the concurrent freezing of millimeter-sized test water droplet and condensed water microdroplets using a digital microscope with a rotating head. The iced microdroplets are marked by red dots, which are recognized by the onset of opacity. Scale bars are 130 μm. (G) A schematic showing the freezing of water droplets correlates to condensation frosting including condensation, frost occurrence along edges, and frost propagation. See also Figure S15.

To further probe the impact of condensation, a digital microscope with a rotating head was applied, which facilitates that in one field-of-view both millimeter-sized water droplets and micrometer-sized condensates can be observed. The time-lapse images revealing the very moment of the freezing of a water droplet on petal-like surface during INT test are shown in Figure 5. The corresponding process was recorded in Video S1. Before the freezing of the water droplet, the surrounding condensed microdroplets started to freeze, which were recognized by the rapid onset of opacity. Here, the frozen microdroplets are marked by red dots. Moreover, they were found to be initiated along edges and propagated toward the center for glass, PDMS, and superhydrophobic surfaces (Figure S15). Notably, PDMS and superhydrophobic surfaces exhibit dropwise condensation. And glass surface shows filmwise condensation, where there is only frozen film instead of frozen microdroplets. Finally, the frozen microdroplets, i.e., frost, on petal-like surface attacked the test droplet and turned it iced (Figure 5F).

Video S1. Concurrent freezing of millimeter-sized water droplet and condensed water microdroplets, related to Figure 5

Additionally, the dependence of INT on cooling rate was measured, and the results are shown in Figure S16. As cooling rate increases from 0.5°C to 4.0ºC min^−1^, the INT for PDMS and superhydrophobic surfaces decreases fast. For instance, the INT for PDMS surface at 0.5°C min^−1^ is −17.8°C, which becomes −29.8°C at 4.0°C min^−1^. And the INT for gecko-like surface at 0.5°C and 4.0ºC min^−1^ is −12.5 and −21.1°C, respectively. On the opposite, the INT for glass surface kept around −2.6°C regardless of what cooling rate was used. The reason is that frost propagation through a condensed film is very fast, whereas the propagation within a network of condensed microdroplets is time-dependent,^49^ which is evident in Figure S15. Thereby, the freezing behavior of water droplets in condensation condition is totally different from that in non-condensation condition. Herein, it strongly correlates to condensation frosting including condensation, frost occurrence at edges, and frost propagation (Figure 5G).

**Figure 6:**
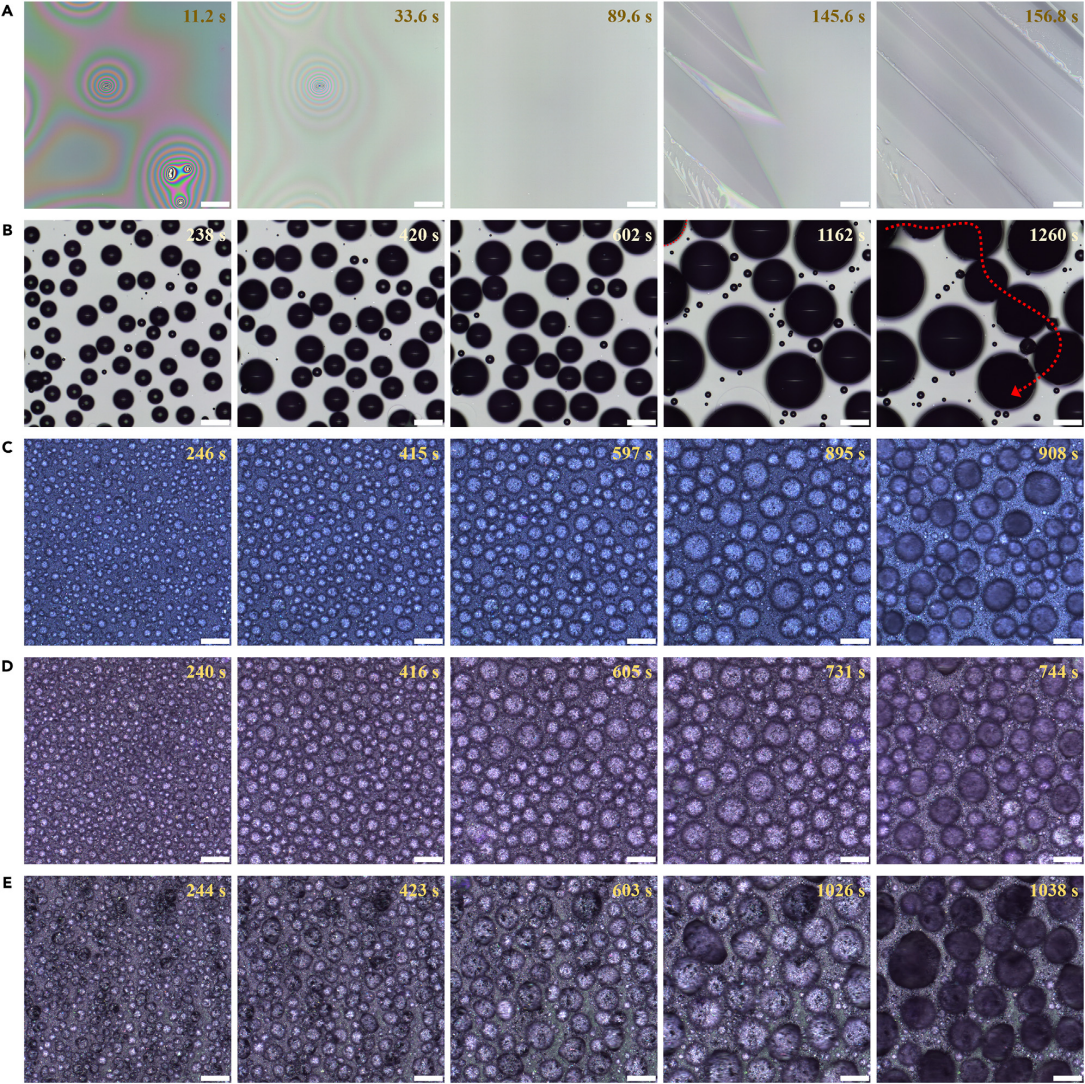
Observation of condensation and frost propagation (A–E) Time-lapse images of condensation and frost propagation on (A) plasma-treated glass surface, (B) PDMS, (C) gecko-like, (D) petal-like, and (E) lotus-like surfaces under a constant cooling rate of 1°C min^−1^. Frost occurs in the last two frames for glass and PDMS surfaces. Only one iced microdroplet (outlined by a red dash curve) shown in the field-of-view at 1,162 s on PDMS surface. Almost all the microdroplets within the field-of-view in the last frames are frozen on three superhydrophobic surfaces. Scale bars are 100 μm.

To this end, condensation and frost propagation on all the five surfaces were investigated (Figure 6). The check positions were the center of surfaces having sizes of 75 × 25 mm, where water droplets were dripped for INT test. Real-time images were taken by the laser scanning confocal microscope, which began at 0°C. Ambient conditions and cooling rate were same to those for INT test. For glass surface, filmwise condensation is manifest (Figure 6A). Condensed microdroplets were clearly observed on PDMS (Figure 6B), gecko-like (Figure 6C), petal-like (Figure 6D), and lotus-like (Figure 6E) surfaces. With increasing time, microdroplets grew and coalesced, and subsequently new ones were born. Finally, condensed film and microdroplets got frozen, which occurred at different time for different surfaces in accordance with their respective INT. It is worth noting that there was no sliding off or jumping removal of condensates.

**Figure 7:**
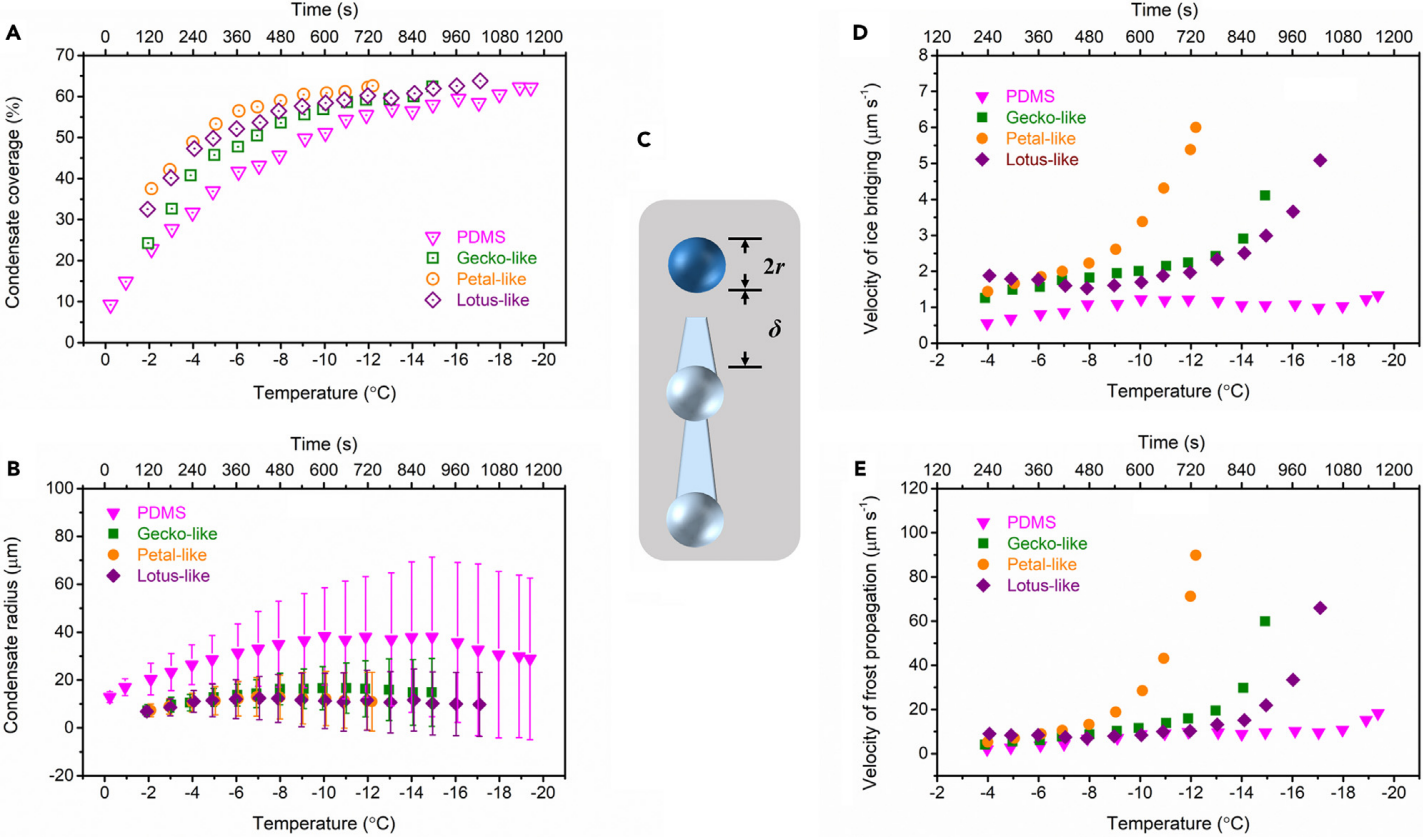
Measurement of condensation and frost propagation (A) Condensate coverage evolution and (B) condensate radius evolution with time/temperature on PDMS, gecko-like, petal-like, and lotus-like surfaces. Data are represented as mean ± SD. (C) A schematic to ice bridging. (D and E) The dependence of (D) velocity of ice bridge growth and (E) velocity of frost propagation on time/temperature for PDMS, gecko-like, petal-like, and lotus-like surfaces. See also Figures S17–S22.

Furthermore, condensate coverage (*ϕ*) and condensate radius (*r*) for PDMS and superhydrophobic surfaces were measured. Microdroplets of radius >3 μm were counted by ImageJ software. The condensate coverage evolution is shown in Figure 7A. Generally, it is divided into four stages: *ϕ* < 30% for separate growth of microdroplets; 30% < *ϕ* < 55% for microdroplet growth dominated by coalescence; a platform at *ϕ* ≈ 55% as a result of the balance between *ϕ* increase by condensation and *ϕ* decrease by coalescence; and *ϕ* > 55% due to the mushrooming of new microdroplets.^50^^,^^51^ Here, the platform moved to *ϕ* ≈ 60%. This is because the condensation was conducted with decreasing temperature, which was under some sub-zero temperature elsewhere.^52^^,^^53^ Additionally, the microdroplets in the first stage on superhydrophobic surfaces were too tiny to check. The condensate radius evolution is shown in Figure 7B. The microdroplets on PDMS surface are always larger than those on superhydrophobic surfaces, which is in agreement with early studies.^54^^,^^55^^,^^56^ The radius growth with time (*t*) can be fitted by the power law, i.e., *r* ∼ *t*^*α*^. For PDMS and superhydrophobic surfaces in the second stage, *α* was calculated to be 0.3–0.5 (Figure S17), which is smaller than early reports of 0.75–1.0,^29^ owing to the cooling procedure applied here.

Notably, frost did not occur in the field-of-view until *ϕ* was larger than 60% for PDMS and superhydrophobic surfaces. It is suggested that the frost propagation requires a close network of microdroplets. Moreover, the velocities of frost propagation differ among these surfaces. For PDMS surface (Figure 6B), the propagation route was marked by a red dash line, from which a velocity around 12 μm s^−1^ is got. For three superhydrophobic surfaces, the whole field-of-view was frozen in one scanning interval (Figures 6C–6E), resulting in velocities of >60 μm s^−1^. In addition, looking at the frost propagation near edges, the velocity was 41.6 ± 13.7 μm s^−1^ around −14.5°C for lotus-like surface (Figure S18) and was 8.1 ± 2.5 μm s^−1^ around −13.2°C for PDMS surface (Figure S19). Hence, frost propagates from edges toward the center with an accelerating speed. And frost propagation is both surface-sensitive and time-dependent.

The dominant mechanism of frost propagation is ice bridging.^49^^,^^57^ It is evident that frost propagated from iced microdroplets to neighboring unfrozen ones in a chain reaction by interconnected ice bridges on PDMS surface (Figure 6B and S15C). Despite rough textures, ice bridging was also observed on superhydrophobic surfaces (Figure S20). The velocity of ice bridge growth (*v*_*b*_) is as below:^58^^,^^59^

(Equation 14) $$v_{b}=\beta \frac{D}{\rho_{i}\bar{R}T_{w}}\frac{p_{s,l}-p_{s,i}}{\delta }\text{,}$$

where *β* is a geometric parameter that depends on the morphology of ice bridges, *D* is the diffusivity of water vapor in air, *ρ*_*i*_ is the density of ice, $\bar{R}$ = 461.5 J (kg·K)^−1^ is the gas constant of water vapor, *T*_*w*_ is the wall (surface) temperature, *δ* is the edge-to-edge spacing (Figure 7C), *p*_*s,l*_ is the saturation pressure of the liquid microdroplet, and *p*_*s,i*_ is the saturation pressure of the iced microdroplet.^59^ Moreover, *D* has the form:^60^^,^^61^

(Equation 15) $$\frac{D}{D_{0}}={\left(\frac{T}{T_{0}}\right)}^{1.75}\text{,}$$

where *D*_*0*_ is 0.220 cm^2^ s^−1^ at *T*_*0*_ of 273.15 K. *δ*, as a function of temperature, was gauged by the built-in LMeye7 software of the laser confocal scanning microscope, and the data are shown in Figure S21. To find out *β*, we measured the *v*_*b*_ for PDMS surface (Figure S22), which was 1.07 ± 0.12 μm s^−1^ between −14°C and −15°C. Thus, *β* of 4.0 is got. Subsequently, the dependence of *v*_*b*_ on temperature for PDMS and superhydrophobic surfaces was drawn in Figure 7D.

As seen from Figure 7C, frost propagation involves both the build-up of ice bridges and the freezing of liquid microdroplets. And the latter is a much faster process, which needs negligible time in comparison to ice bridging. In this case, the velocity of frost propagation (*v*_*p*_) can be expressed as follows^58^:

(Equation 16) $$v_{p}\approx \left(1+\frac{2r}{\delta }\right)v_{b}\text{.}$$

The plots of *v*_*p*_ versus temperature for PDMS and superhydrophobic surfaces are shown in Figure 7E. For all surfaces, *v*_*p*_ keeps low for a long time before an exponential rise that presages the frost occurrence in the field-of-view. And the calculated *v*_*p*_ values at the last dots are all above 60 μm s^−1^ for three superhydrophobic surfaces, which are consistent with the experimental results (Figures 6C–6E). For PDMS surface, the calculated *v*_*p*_ at the last dot is 18 μm s^−1^, which is comparable to its experimental counterpart of 12 μm s^−1^ (Figure 6B). It is worth noting that *v*_*p*_ is decisive for frost propagation, and the time when *v*_*p*_ goes a steep rise determines the INT.

**Figure 8:**
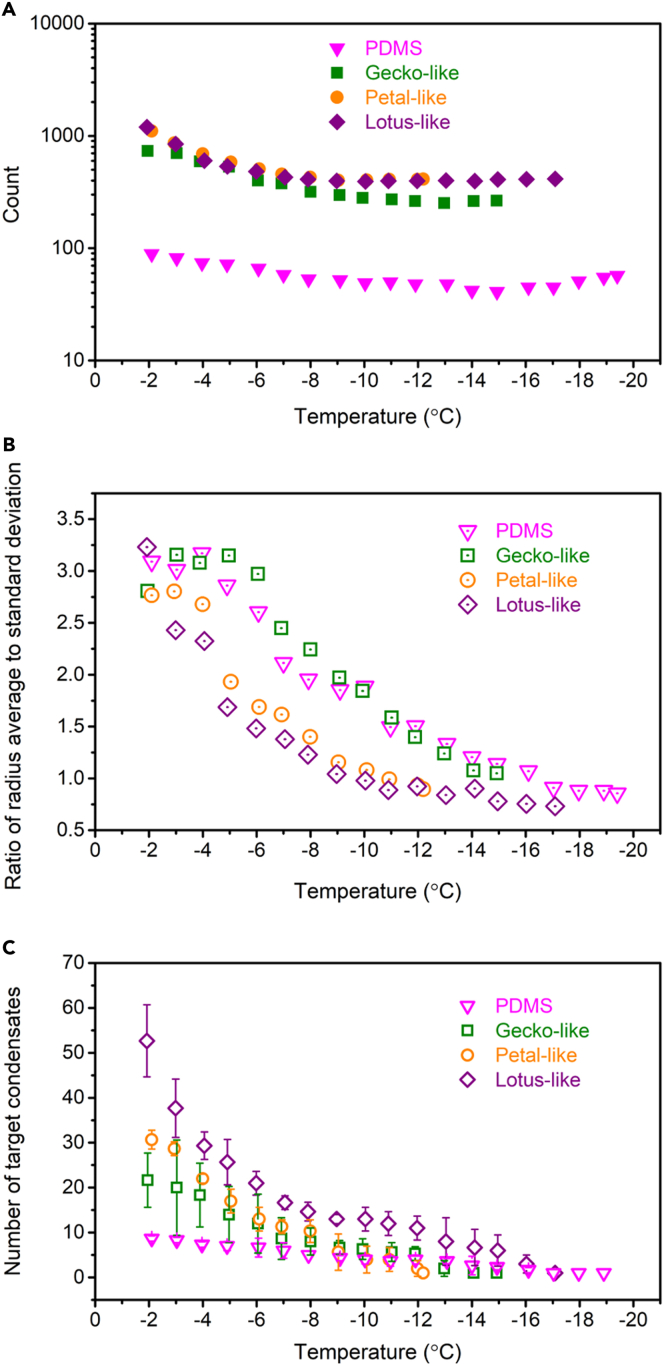
Factors affecting frost propagation velocity (A) Number of condensates on an area of 750 μm × 750 μm with decreasing temperature on PDMS, gecko-like, petal-like, and lotus-like surfaces. Condensates with radii larger than 3 μm were counted. (B) Change of the ratio of condensate radius average to standard deviation with temperature for PDMS, gecko-like, petal-like, and lotus-like surfaces. (C) Condensate number evolution by coalescence during cooling on PDMS, gecko-like, petal-like, and lotus-like surfaces. Three large microdroplets upon freezing were chosen as observation targets for each surface. Data are represented as mean ± SD. See also Figure S23.

It is obvious that *v*_*p*_ differences among surfaces result from their different *δ* and *r*, in which *δ* is more important. If condensed microdroplets are mono- and uniformly dispersed, we have

(Equation 17) $$\delta =\frac{1}{\sqrt{n}}-2r\text{,}$$

where *n* is the areal density of microdroplets. Thus, *δ* increases with decreasing *n* or decreasing *r*. While possessing larger *r* than superhydrophobic surfaces (Figure 7B), PDMS surface exhibits far smaller condensate density (Figure 8A). Around −2°C, the densities are 1.58 × 10^8^, 1.31 × 10^9^, 1.97 × 10^9^, and 2.13 × 10^9^ m^−2^ for PDMS, gecko-like, petal-like, and lotus-like surfaces, respectively, which is consistent with the previous finding that the rougher surfaces became, the more condensates were formed.^52^^,^^62^^,^^63^

However, the mono-dispersity assumption only holds for a short time as the ratio of radius average to standard deviation decreases fast (Figure 8B). Providing it is a binary-dispersity system (radius *r*_*1*_ and radius *r*_*2*_, *r*_*1*_ = *b* × *r*_*2*_), the δ change has the following form if both the condensate coverage and the average of two microdroplets are fixed:

(Equation 18) $$\frac{\delta_{binary}}{\delta_{mono}}∼\frac{{2\bar{r}+\delta }_{binary}}{{2\bar{r}+\delta }_{mono}}=\frac{\sqrt{2\left(1+b^{2}\right)}}{\left(1+b\right)}\text{,}$$

where $\bar{r}$ is the average of *r*_*2*_ and *r*_*1*_. Thereby, *δ* monotonically increases with enlarging the gap between two microdroplet sizes. For lotus-like surface, the ratio of radius average to standard deviation plummeted at the very start, after which it remained the smallest until microdroplets turned iced (Figure 8B). To explore the reason, three large microdroplets upon freezing (Figure S23) were selected to monitor their evolution during cooling. It turned out that nearly 53 microdroplets were coalesced into one on lotus-like surface, which are 9, 22, and 31 for PDMS, gecko-like, and petal-like surfaces, respectively (Figure 8C). Therefore, the lotus-like topography facilitates the coalescence of condensates,^64^^,^^65^ which serves an effective way to enlarge *δ*, resulting in the lower INT for lotus-like surface than gecko-like and petal-like surfaces.

## Discussion

Herein, a huge INT gap up to 18°C was discerned among different kinds of superhydrophobic surfaces using different test environments. In non-condensation condition, the freezing of water droplets on surfaces is caused by heterogeneous nucleation at droplet-surface interface, which instead strongly correlates to condensation frosting in condensation condition. Owning to such distinct freezing mechanisms, the roles of surface chemistry and surface topography of superhydrophobic surfaces in anti-icing vary significantly. In non-condensation condition, both surface chemistry and surface topography contribute to anti-icing. The former raises the energy barrier for ice nucleation by employing low-surface-energy materials, and the latter lowers the nucleation kinetics by reducing the contact between droplets and surfaces. In condensation condition, the role of surface chemistry remains positive, which defers the frost propagation by forming dropwise condensation. But the role of surface topography becomes complex: on one hand, it speeds up the frost propagation due to the increasing condensate density; on the other hand, it slows down the frost propagation via the fast coalescence of condensates that is evident in lotus-like surface. As a result, low surface energy benefits the anti-icing in both non-condensation and condensation conditions. Nano-sized textures are promising in non-condensation condition, whereas frost-free/frost-delay textures enabling fast coalescence, jumping removal,^29^^,^^45^ or large vapor pressure gradients^66^ have great potential in condensation condition.

### Limitations of the study

We have revealed two different freezing mechanisms of water droplets on superhydrophobic surfaces and thus the role variation of surface chemistry and surface topography in anti-icing. However, we set several preconditions: there was no heat transfer between surfaces and water droplets in non-condensation environment; there was no jumping removal of condensates in condensation environment; and all surfaces were placed horizontally. Therefore, future investigation is needed to explore more situations.

## Resource availability

### Lead contact

Further information and requests for resources should be directed to and will be fulfilled by the lead contact, Masanobu Naito (naito.masanobu@nims.go.jp).

### Materials availability

This study did not generate new unique reagents.

### Data and code availability

• This study did not generate any datasets. All data reported in this paper will be shared by the lead contact upon request.
• This paper does not report original code.
• Any additional information required to reanalyze the data reported in this paper is available from the lead contact upon request.

## Acknowledgments

This work was supported by the 10.13039/501100003382Core Research for Evolutional Science and Technology (CREST) program “Revolution material development by fusion of strong experiments with theory/data science” of the Japan Science and Technology Agency (JST), Japan, under Grant JPMJCR19J3.

## Author contributions

Conceptualization, W.W. and M.N.; methodology, W.W. and M.T.; investigation, W.W. and M.T.; writing—original draft, W.W.; writing—review & editing, M.T. and M.N.; funding acquisition, M.N.; resources, M.T. and M.N.; supervision, W.W. and M.N.

## Declaration of interests

The authors declare no competing interests.

## STAR★Methods

### Key resources table

**Table:** 

REAGENT or RESOURCE	SOURCE	IDENTIFIER
**Chemicals, peptides, and recombinant proteins**		

ZnO tetrapod	AMTEC Co., Ltd.	WZ-0501
Polydimethylsiloxane (PDMS)	Toray Industries, Inc.	HC2100
Ethyl acetate	KANTO	Cat#14029-80
Rhodamine B	WAKO	Cat#180-00132

**Other**		

Glass slides	Matsunami Glass Ind., Ltd.	S7224
Ion bombarder	Vacuum Device	PIB-10
Tabletop scanning electron microscope	Hitachi	Miniscope TM3000
Field emission scanning electron microscope	JEOL	JSM-7001F
Laser confocal scanning microscope	Lasertec	Optelics HYBRID C3
X-ray photoelectron spectroscope	ULVAC-PHI Inc.	Quantera SXM
Contact angle meter	Kyowa Interface Science	401-type
Environmental chamber	ETAC	FX420N
Cooling stage	High Tech Co., Ltd.	10083L
High-speed camera	Photron	Mini AX, FASTCAM
Digital microscope	Olympus Corporation	DSX-1000
Laser confocal fluorescence microscope	Leica	TCS SP5
Dip-coater	Aiden	DC4300
Spin-coater	MIKASA	MS-B150

### Method details

#### Preparation of surfaces

ZnO tetrapods (pana-tetra WZ-0501) were purchased from AMTEC Co., Ltd. (Japan). PDMS (HC2100) was bought from Toray Industries, Inc. (Japan). For gecko-like surface, first a suspension of 9 g ZnO and 1 g PDMS in 60 mL ethyl acetate (EtOAc, 99.5%) was prepared. Afterward, the suspension was spin-coated on glass slides (S7224, Matsunami Glass Ind., Ltd., Japan) with a rotation speed of 200 rpm, a coating time of 30 s, and a repeating number of 2 by a spin-coater (MS-B150, MIKASA, Japan). For petal-like surface, a suspension of 9 g ZnO and 1 g PDMS in 40 mL EtOAc was made. Then, the suspension was spin-coated on glass slides with a rotation speed of 200 rpm, a coating time of 30 s, and a repeating number of 1. As to lotus-like surface, same suspension for petal-like surface was used, which was dip-coated on glass slides with a pull-out speed of 5 mm s^−1^ and a repeating number of 8 by a dip-coater (DC4300, Aiden, Japan). Additionally, for PDMS surface, a solution of 4.5 g PDMS in 24 mL EtOAc was made, which was then spin-coated on glass slides with a rotation speed of 200 rpm, a coating time of 300 s, and a repeating number of 4. After coating, all surfaces were dried at 80°C for 2 h to remove solvent and cure silicone.

#### Plasma treatment of glass slides

To make superhydrophilic surfaces, the as-received glass slides (S7224, Matsunami Glass Ind., Ltd., Japan) were plasma-treated using an ion bombarder (PIB-10, Vacuum Device, Japan) with a discharge current of 10 mA and a process time of 3 min.

#### Morphology and composition characterization

Tabletop scanning electron microscope (SEM, Miniscope TM3000, Hitachi, Japan) and field emission scanning electron microscope (FESEM, JSM-7001F, JEOL, Japan) were used to observe the morphology of ZnO tetrapods, PDMS, and superhydrophobic surfaces. Moreover, laser confocal scanning microscope (Optelics HYBRID C3, Lasertec, Japan) was applied to obtain 3D surface topography of superhydrophobic surfaces. Surface chemical compositions of ZnO tetrapods and superhydrophobic surfaces were tested by X-ray photoelectron spectroscope (XPS, Quantera SXM, ULVAC-PHI Inc., Japan).

#### Wettability characterization

Static contact angle (CA), advancing CA (*θ*_*adv*_), and receding CA (*θ*_*rec*_) were measured using DMs-401-type CA meter (Kyowa Interface Science, Japan), where water droplets of 8 μL were applied. And ambient temperature of 22 ± 1°C and relative humidity (RH) of 52 ± 2% were kept. Each CA test was repeated at least 5 times. Furthermore, water impalement into superhydrophobic surfaces was studied. Water droplets (10 μL) doped with 0.02 wt. % of Rhodamine B were placed on surfaces either at room temperature for 20 min or under cooling to −20°C at 1°C min^−1^ (in non-condensation condition) before removal. Subsequently, 3D distribution of fluorescent dye residue in surfaces was probed by laser confocal fluorescence microscope (TCS SP5, Leica, Germany).

#### Anti-icing performance test

There were two test conditions: non-condensation (Figure S3A) and condensation (Figure S3B) conditions. The non-condensation condition was realized in an environmental chamber (FX420N, ETAC, Japan). Here, the temperature of test surfaces was same to the chamber temperature. With the chamber temperature decreasing from 20°C to −35°C at 1.0°C min^−1^, the chamber RH followed a route shown in Figure S4A. As a result, the chamber temperature was always higher than the corresponding dew point (Figure S4B), which guaranteed a non-condensation test condition. As to the condensation condition, tests were conducted in an experimental stall, where the ambient temperature and RH were maintained at 22 ± 1°C and 52 ± 2%, respectively, leading to a fixed dew point around 11.7°C. Test surfaces were mounted on a cooling stage (10083L, High Tech Co., Ltd., Japan), on which condensation would happen when the surface temperature was lower than the dew point.

To test ice nucleation temperature (INT) under non-condensation condition, test surfaces were first placed in the FX420N chamber at 10°C for 30 min for stabilization. Second, water droplets of 10 μL were dropped on the surfaces. Third, the chamber temperature was decreased at a constant rate of 1.0°C min^−1^. When the water droplets changed from transparent to opaque, the corresponding temperature was taken as the INT. As for INT tests in condensation condition, test surfaces were mounted on the 10083L cooling stage at room temperature (22 ± 1°C). Then, water droplets of 10 μL were dropped on the surfaces. Afterward, the cooling stage was started and the surface temperature was decreased at 10°C min^−1^ before 0°C and at 1.0°C min^−1^ thereafter. Finally, the temperature at which the water droplets turned opaque was recorded as the INT.

Freezing delay time (FDT) was also measured. In non-condensation condition, test surfaces were first put in the FX420N chamber at a setting temperature (below 0°C) for 30 min. Then, water droplets of 10 μL were dropped on the surfaces, which became opaque after a while. The stretch was taken as the FDT corresponding to the setting temperature. In condensation condition, same procedure was adopted. However, once dripped on surfaces, water droplets instantly got frozen. Therefore, it failed to test FDT in condensation condition.

Deionized water (18 MΩ cm at 25°C) was used in both anti-icing and wettability tests. And a high-speed camera (Mini AX, FASTCAM, Photron, Japan) was applied to monitor the freezing of water droplets. The INT for each surface under either non-condensation condition or condensation condition was tested at least 20 times. Every FDT test corresponding to one setting temperature in non-condensation condition was repeated at least 5 times.

#### Condensation test

On one hand, a digital microscope having a rotating head (DSX-1000, Olympus Corporation, Japan) was utilized to acquire images containing both millimeter-sized water droplets and micrometer-sized condensed microdroplets in one field-of-view. To observe condensed microdroplets near the contact line of millimeter-sized water droplets, the head was titled around 40°. On the other hand, laser confocal scanning microscope (Optelics HYBRID C3, Lasertec, Japan) was applied to monitor condensation growth, frost occurrence, and frost propagation. The ambient conditions for condensation were 22 ± 1°C and 52 ± 2% of RH. Test surfaces were mounted on the 10083L cooling stage, and the surface temperature was decreased at a constant rate of 1°C min^−1^. Apparently, the test conditions were same to those for INT test in condensation condition.

### Quantification and statistical analysis

The error bars in Figure S2 represent the standard deviation (SD) obtained using five independent measurements. The error bars in Figures 3A and 4A represent the SD obtained using twenty independent measurements. The error bars in Figures 7B, S17, and S21 represent the SD obtained using tens of or hundreds of condensates depending on type of surfaces. The error bars in Figure 8C represent the SD obtained using three independent measurements. Mean and SD for velocity of frost propagation on lotus-like surface was obtained using seven independent measurements. Mean and SD for velocity of frost propagation on PDMS surface was obtained using six independent measurements. Mean and SD for velocity of ice bridge growth on PDMS surface was obtained using four independent measurements.
