# Supplementary material for: Role variability of surface chemistry and surface topography in anti-icing performance
Source: iScience. 2024 Sep 28;27(11):111039. doi: 10.1016/j.isci.2024.111039 (PMC11700627; doi:10.1016/j.isci.2024.111039)
Supplement: Document S1. Figures S1–S23 [file mmc1.pdf]

iScience, Volume *27*

Supplemental information

Role variability of surface chemistry

and surface topography in anti-icing performance

Wei Weng, Mizuki Tenjimbayashi, and Masanobu Naito

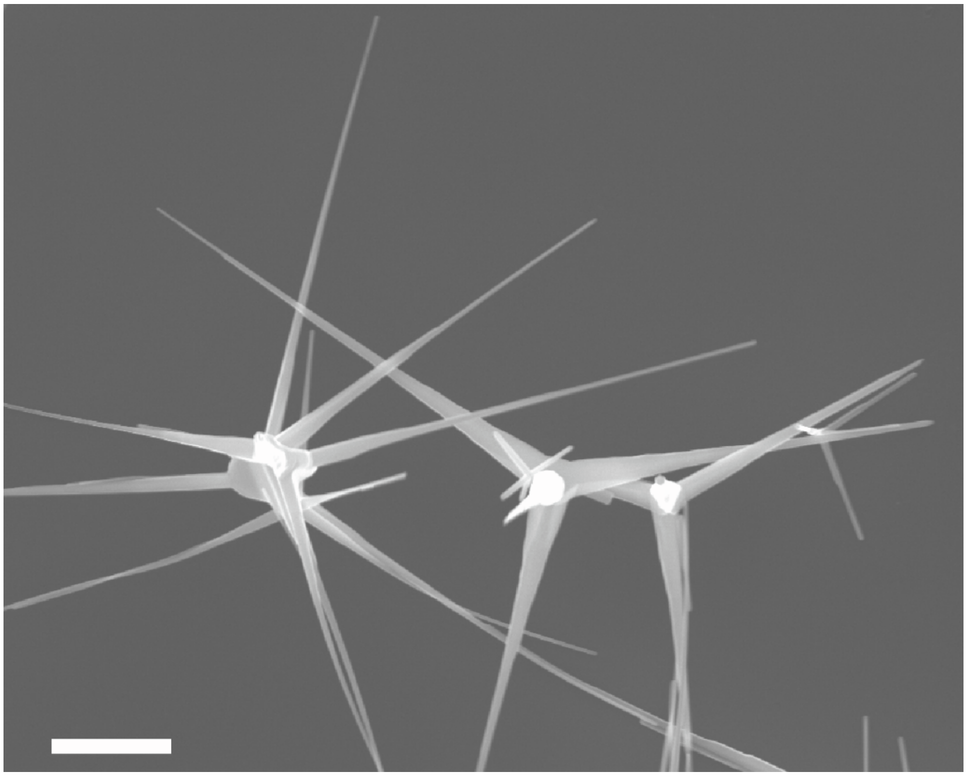

Figure S1. Image of ZnO tetrapods, related to Figure 2.
A high-magnification SEM image of micrometer-sized ZnO tetrapods. Scale bar is 3 μm.

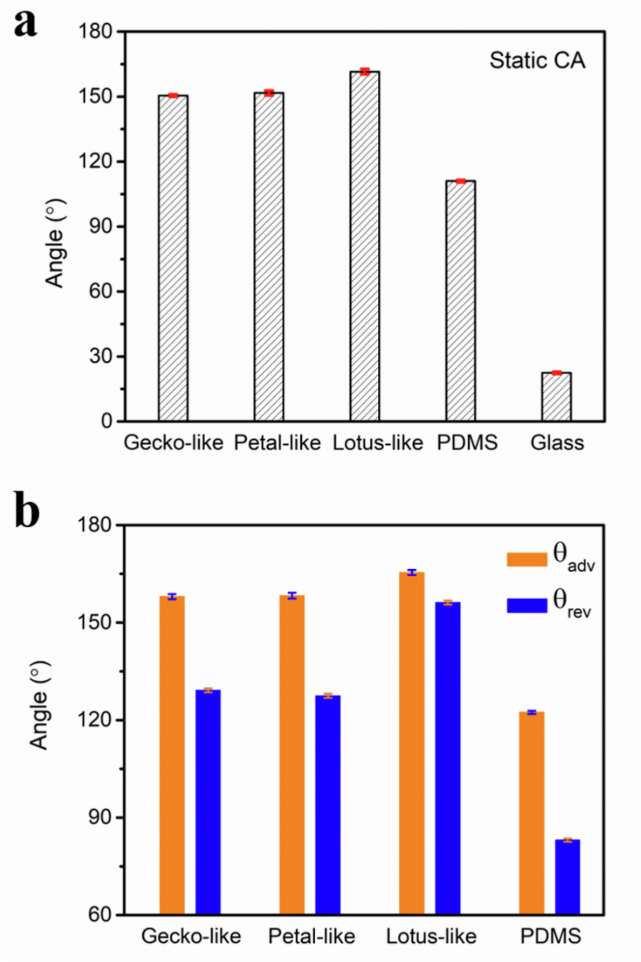

Figure S2. Wettability of surfaces, related to Figure 2.
(a) Static water CAs of glass, PDMS, gecko-like, petal-like, and lotus-like surfaces. Data are represented as mean ± SD. (b) Advancing CA (θ_{adv}) and receding CA (θ_{rec}) of PDMS, gecko-like, petal-like, and lotus-like surfaces. Data are represented as mean ± SD.

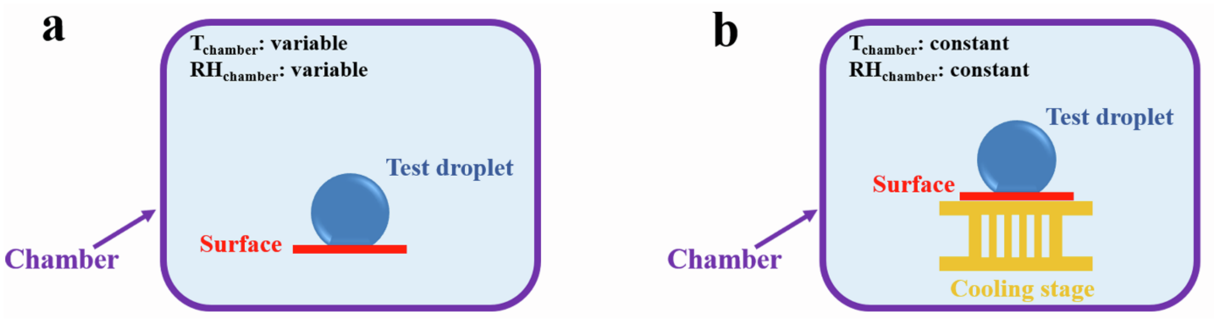

Figure S3. Schematics of non-condensation and condensation test setup, related to STAR Methods. Illustrations of anti-icing test in (a) non-condensation condition and (b) condensation condition.

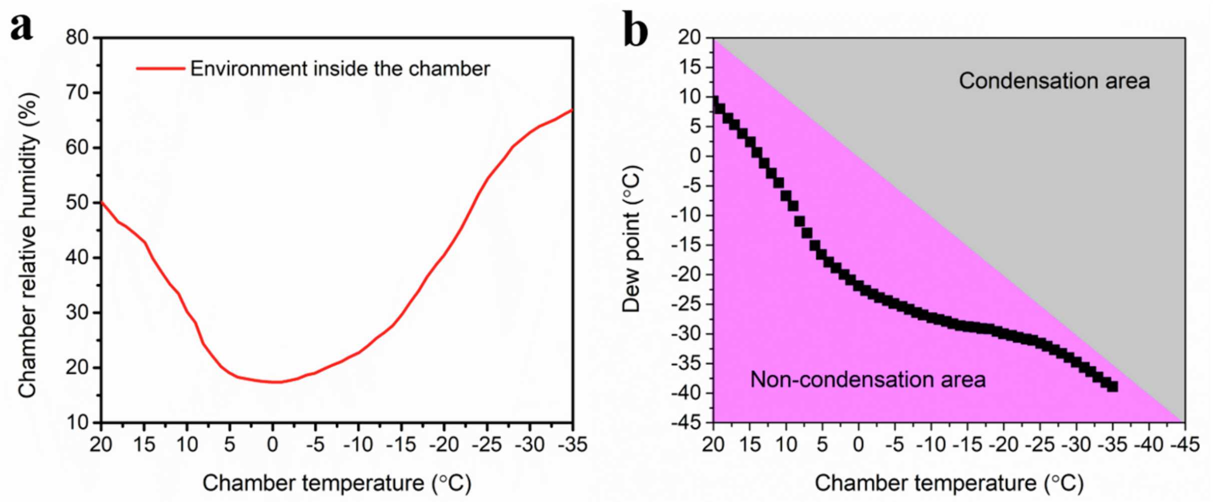

Figure S4. Test environment in non-condensation condition, related to STAR Methods.
(a) Dependence of relative humidity on temperature inside the environmental chamber. (b) Demonstration that the chamber temperature is always higher than the corresponding dew point, guaranteeing a non-condensation test condition.

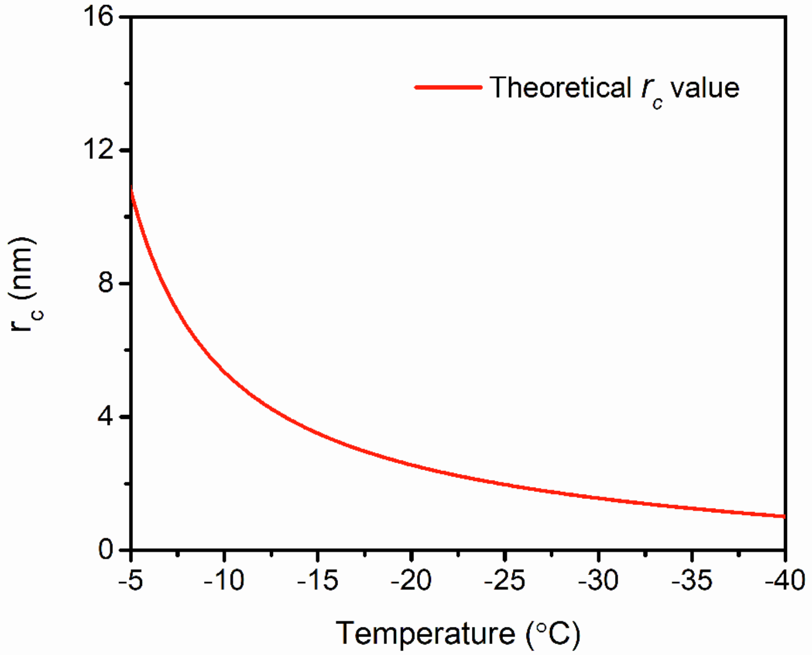

Figure S5. r_c value, related to Figure 3.
Theoretical r_c value as a function of temperature.

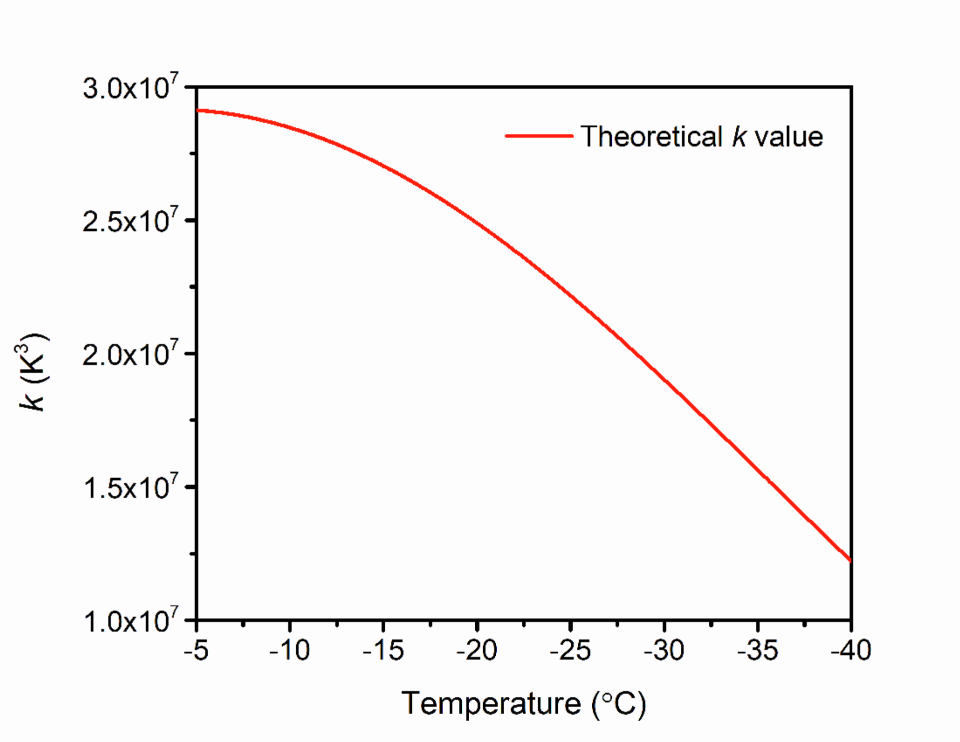

Figure S6. *k* value, related to Figure 3.
Theoretical *k* value as a function of temperature.

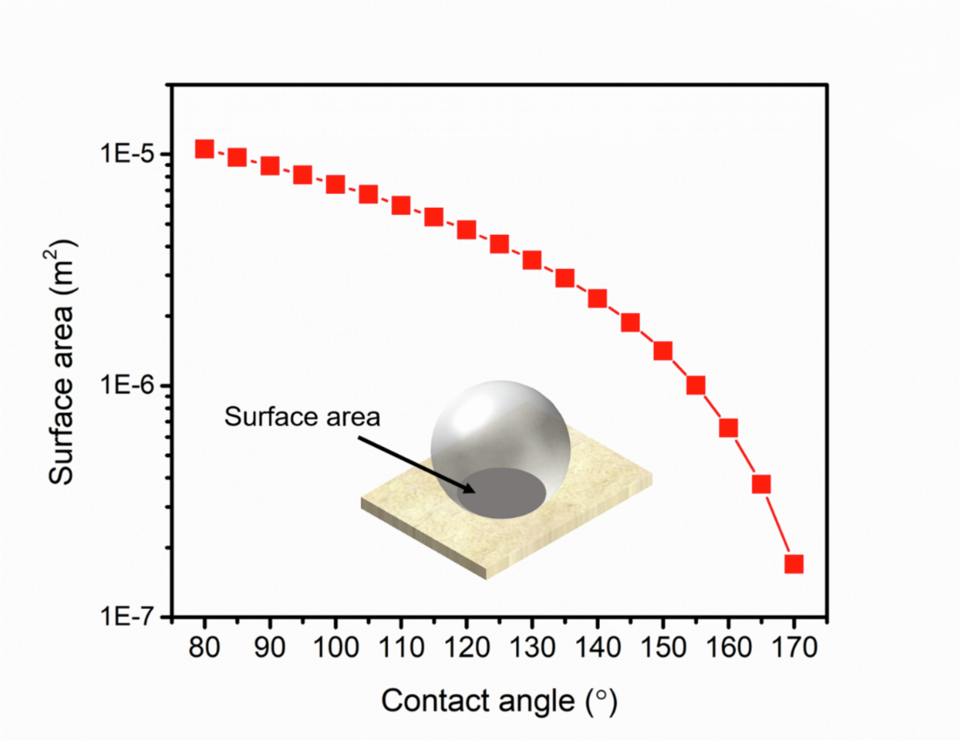

Figure S7. Contact area as a function of contact angle, related to Figure 3.
Surface (contact) area between water droplets (10 μL) and smooth surfaces as a function of contact angle. Inset is a schematic of a water droplet regarded as a spherical cap sitting on a smooth surface.

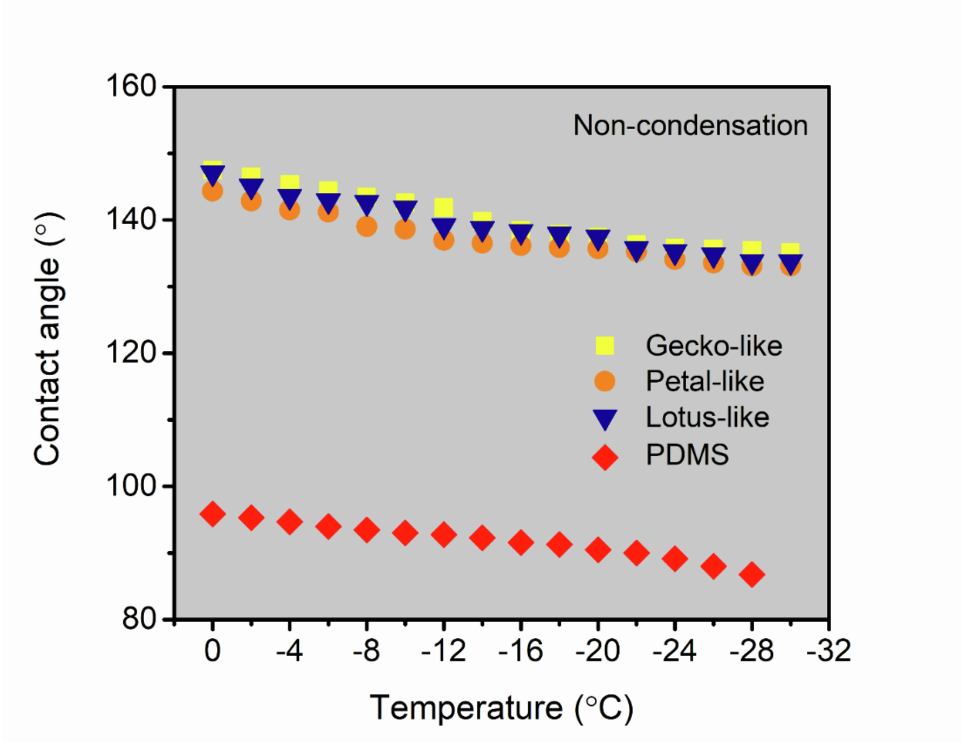

Figure S8. Wettability of surfaces at sub-zero temperatures, related to Figure 3.
CAs of water droplets on PDMS, gecko-like, petal-like and lotus-like surfaces with decreasing temperatures in non-condensation condition.

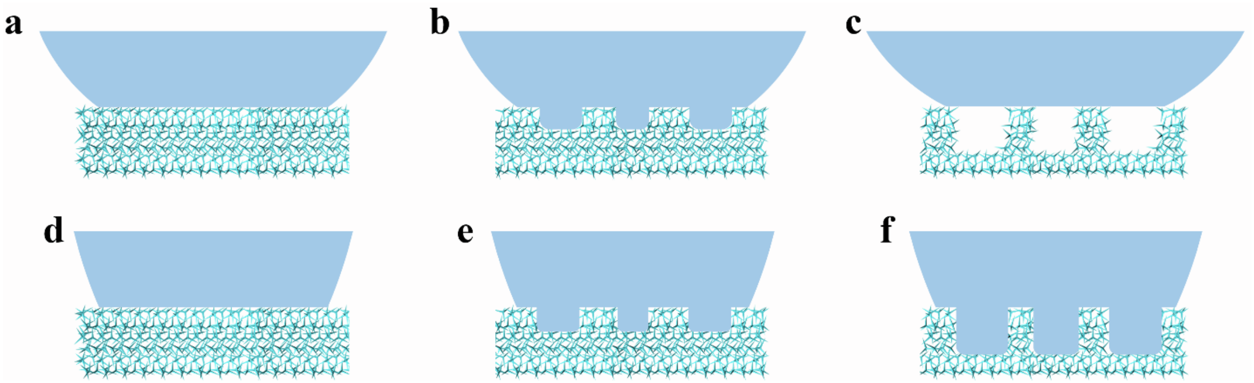

Figure S9. Schematics of water-surface interface, related to Figure 3.
Schematics of water droplets on (a) gecko-like, (b) petal-like, and (c) lotus-like surfaces at room temperature. Schematics of water droplets on (d) gecko-like, (e) petal-like, and (f) lotus-like surfaces at sub-zero temperatures before icing.

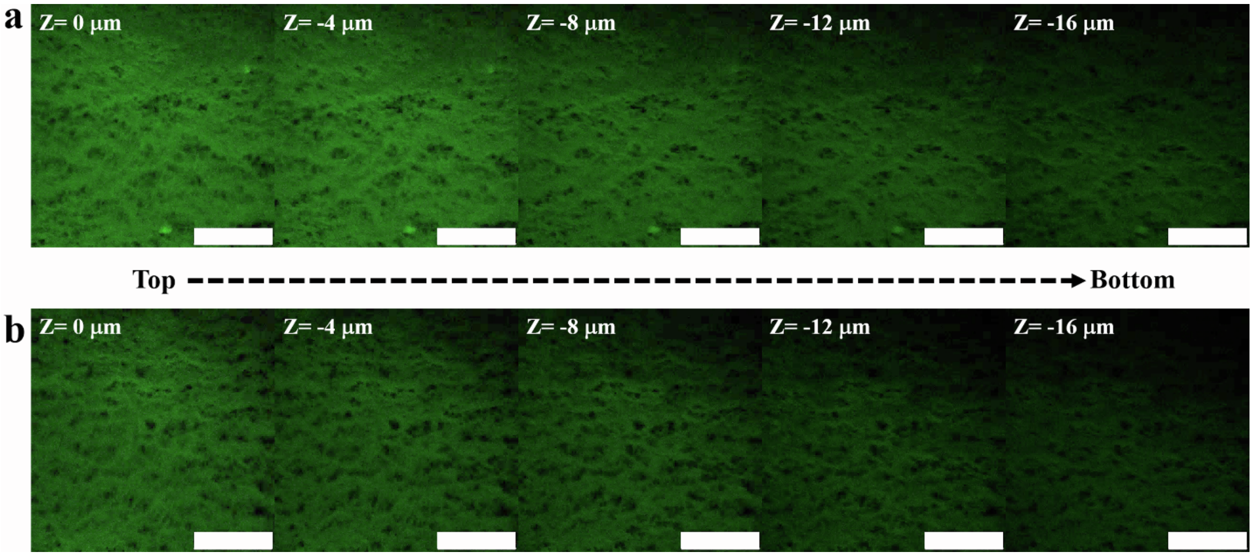

Figure S10. Water impalement characterization for lotus-like surface, related to Figure 3.
3D distribution of fluorescent dye residue in lotus-like surface. Water droplets doped with a slight amount of Rhodamine B were placed on surfaces either at room temperature for 20 min (the lower part) or being cooled to -20 ºC at a rate of 1 ºC min^{-1} (the upper part) before removal. Scale bars are 250 μm.

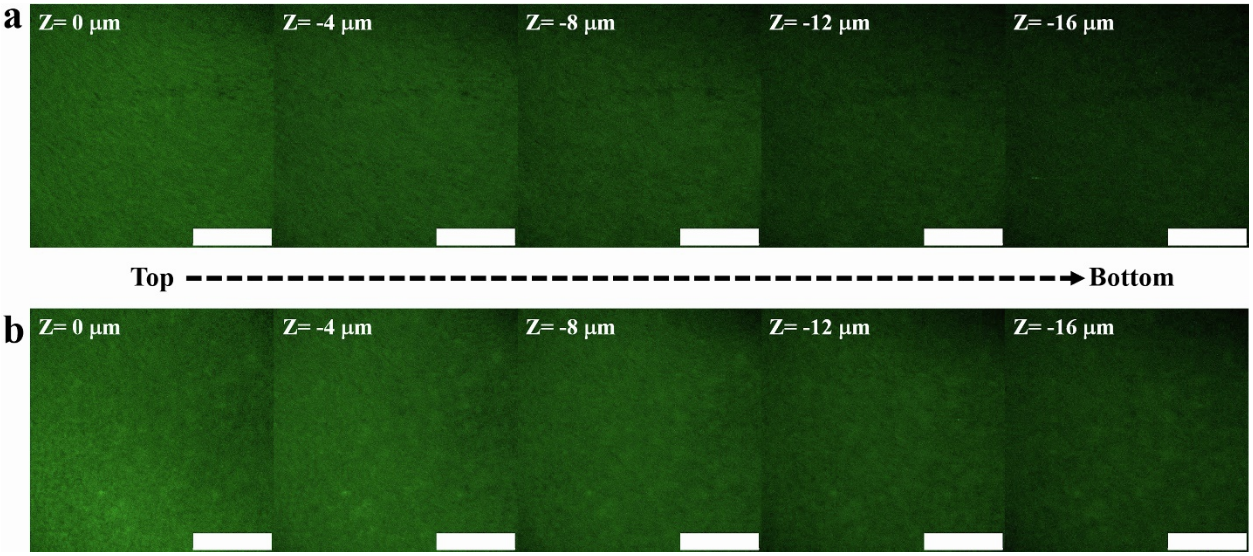

Figure S11. Water impalement characterization for gecko-like surface, related to Figure 3.
3D distribution of fluorescent dye residue in gecko-like surface. Water droplets doped with a slight amount of Rhodamine B were placed on surfaces either at room temperature for 20 min (the lower part) or being cooled to -20 ºC at a rate of 1 ºC min^{-1} (the upper part) before removal. Scale bars are 250 μm.

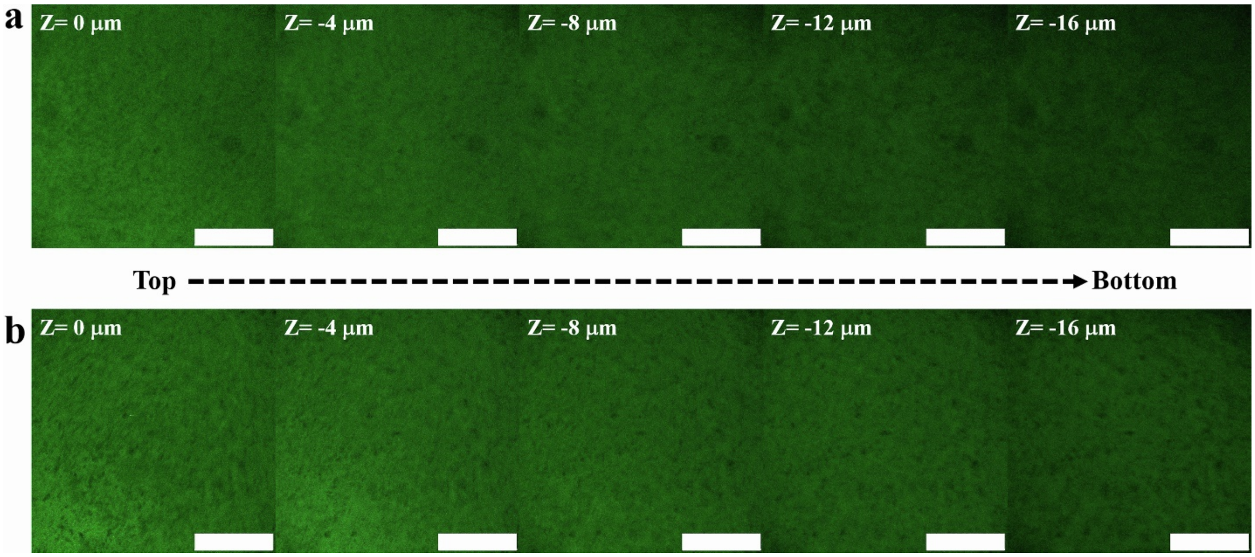

Figure S12. Water impalement characterization for petal-like surface, related to Figure 3.
3D distribution of fluorescent dye residue in petal-like surface. Water droplets doped with a slight amount of Rhodamine B were placed on surfaces either at room temperature for 20 min (the lower part) or being cooled to -20 ºC at a rate of 1 ºC min^{-1} (the upper part) before removal. Scale bars are 250 μm.

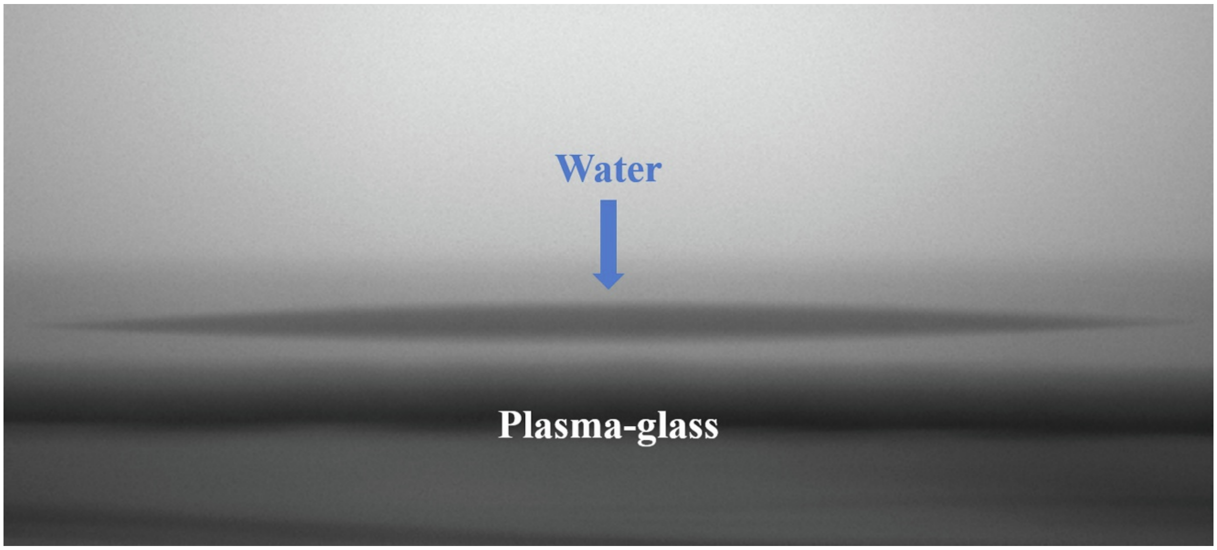

Figure S13. Plasma-glass characterization, related to Figure 4.
Static CA test of a water droplet on plasma-treated glass surface that shows a superhydrophilic property.

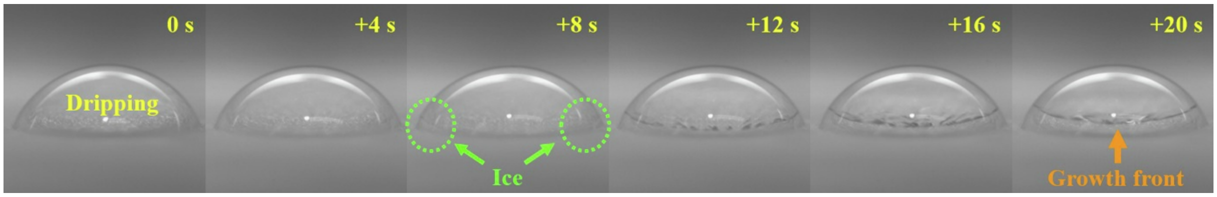

Figure S14. Water droplet freezing observation, related to Figure 4.
Snapshots of the freezing of a water droplet on lotus-like surface that had been kept at -10 ºC for 30 min in condensation condition. 0s refers to the time when the droplet was dripped on the surface.

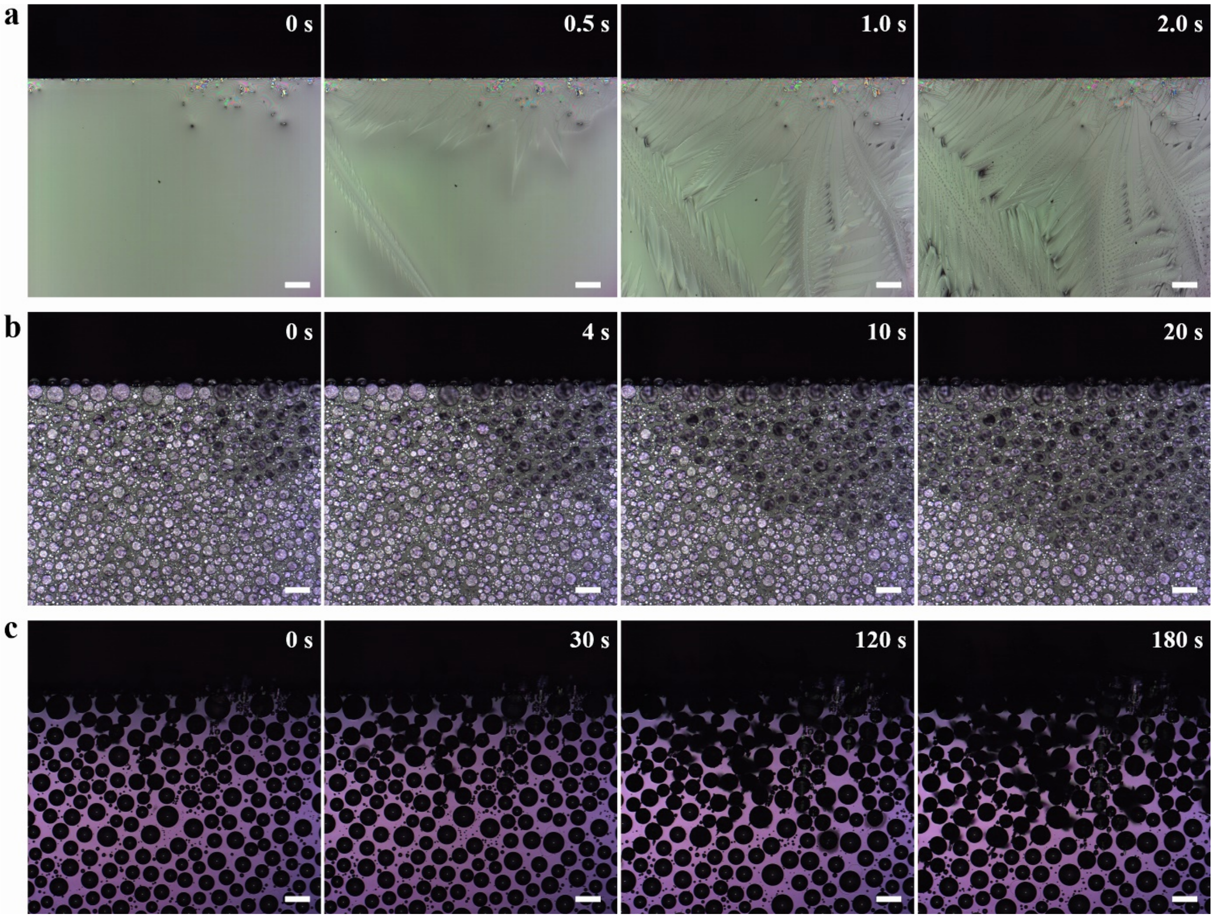

Figure S15. Frost occurrence along edges, related to Figure 5.
Time-lapse images showing frost occurrence along edges on (a) plasma-treated glass surface, (b) lotus-like surface, and (c) PDMS surface in condensation condition. Scale bars are 250 μm.

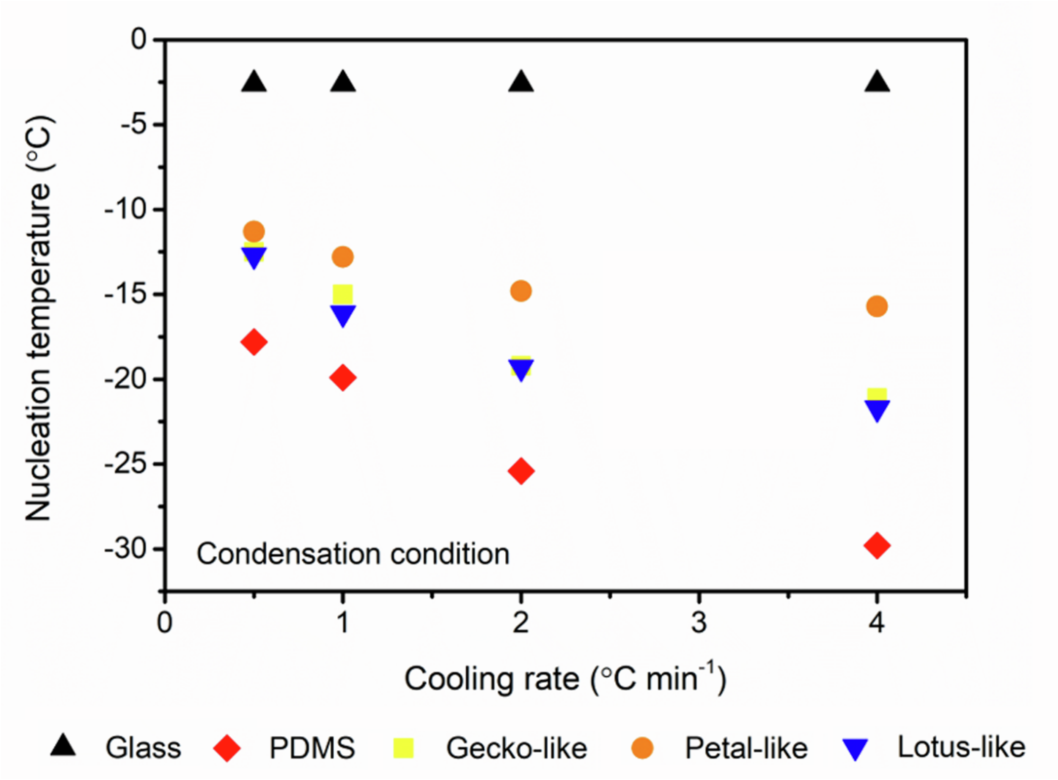

Figure S16. INT dependence on cooling rate, related to Figure 4.
Dependence of ice nucleation temperature on cooling rate for plasma-treated glass, PDMS, gecko-like, petal-like, and lotus-like surfaces, which was tested in condensation condition.

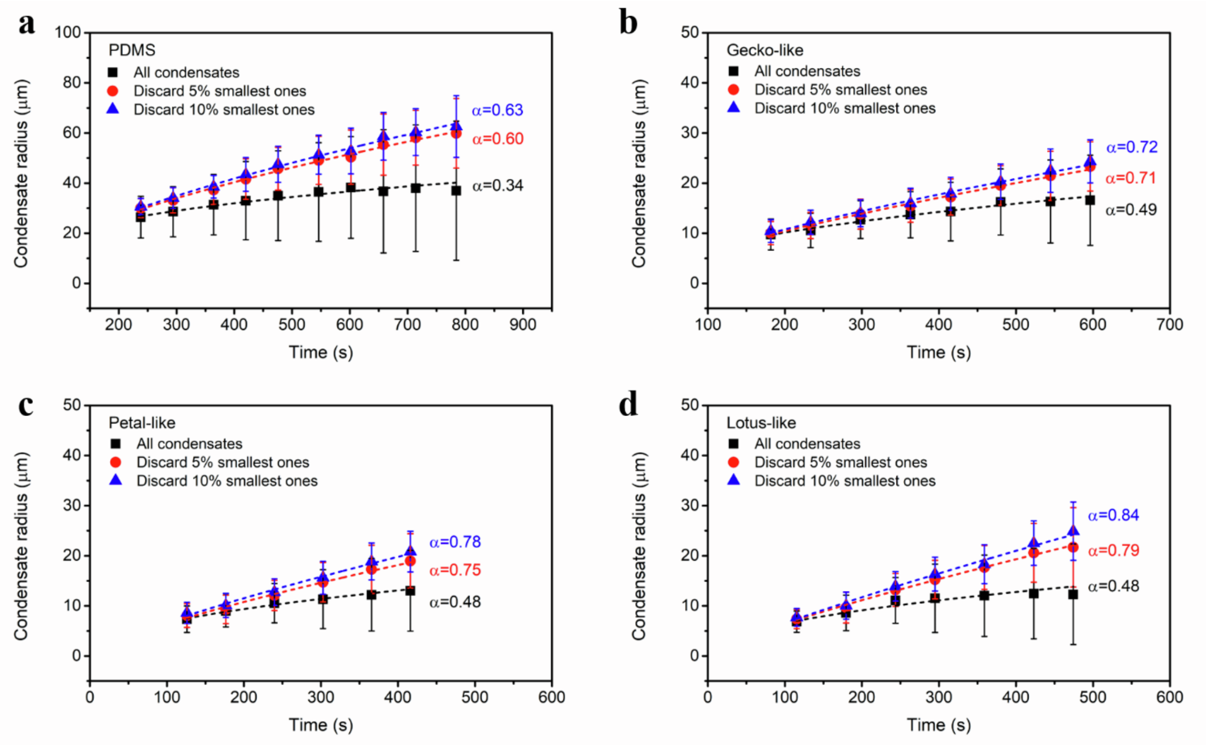

Figure S17. Condensate radius growth, related to Figure 7.
Condensate radius evolution with time for (a) PDMS, (b) gecko-like, (c) petal-like, and (d) lotus-like surfaces in condensation condition. Data are represented as mean ± SD. The radius growth with time was fitted by the power law, $r \sim t^{\alpha}$. Here, only the second stage of condensate growth is shown.

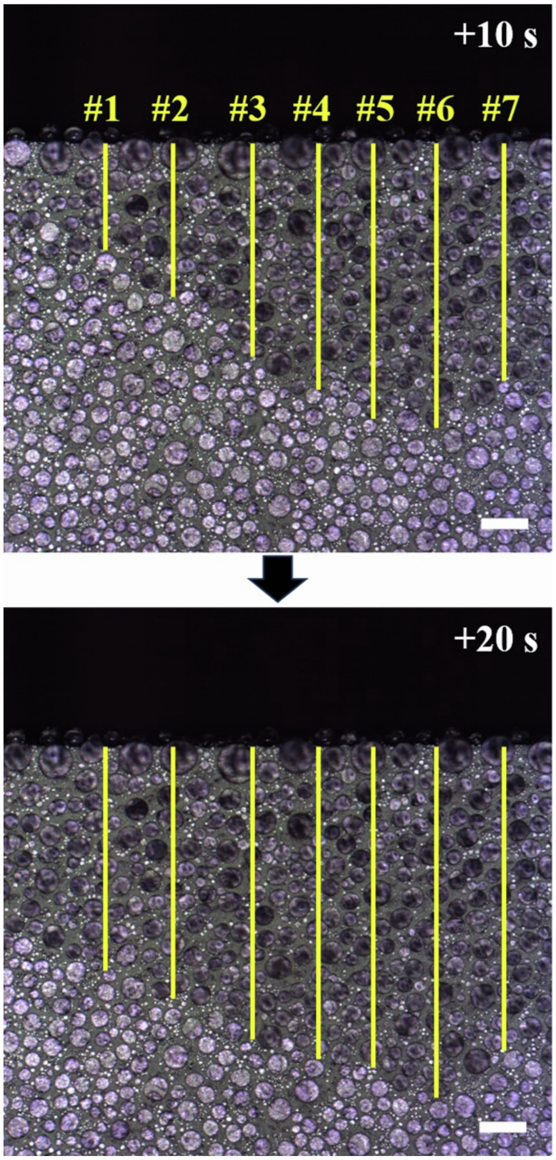

Figure S18. Frost propagation velocity test, related to Figure 7.
Time-lapse images used for measuring the velocity of frost propagation near edges for lotus-like surface in condensation condition. Scale bars are 250 μm.

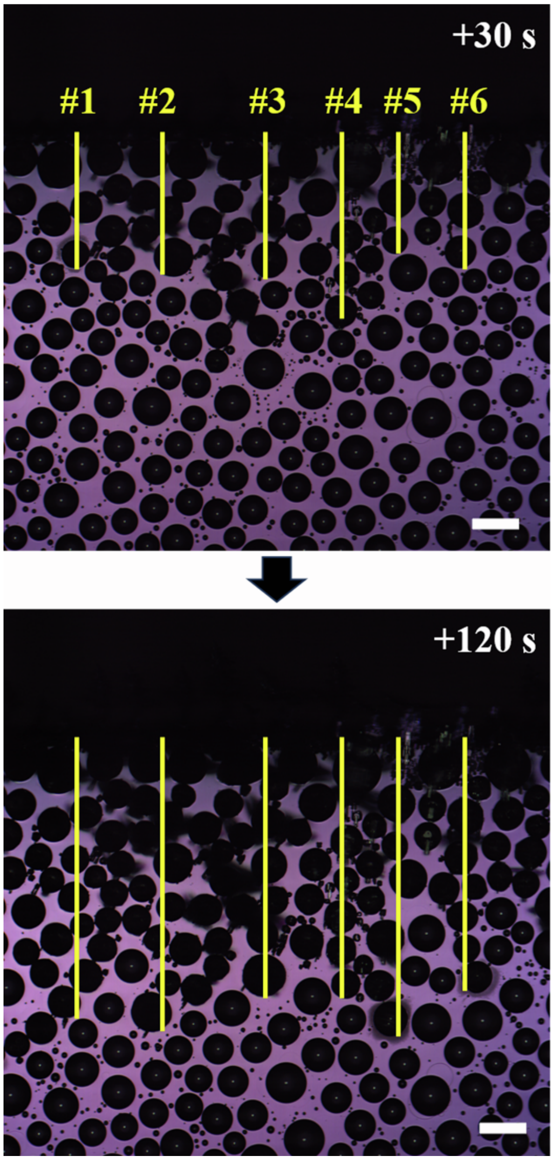

Figure S19. Frost propagation velocity test, related to Figure 7.
Time-lapse images used for measuring the velocity of frost propagation near edges for PDMS surface in condensation condition. Scale bars are 250 μm.

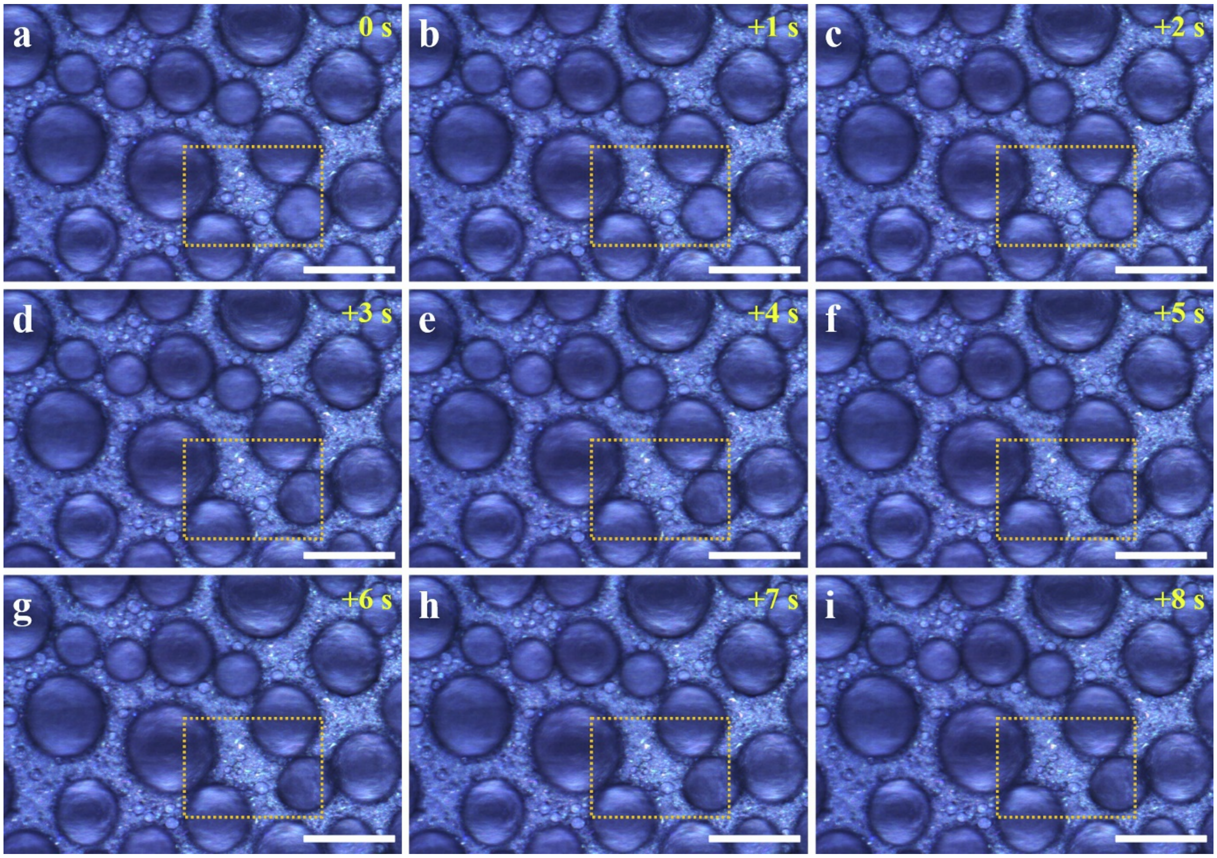

Figure S20. Ice bridging observation, related to Figure 7.
Time-lapse images showing ice bridging in the dash-line square on gecko-like surface in condensation condition. Scale bars are 100 μm.

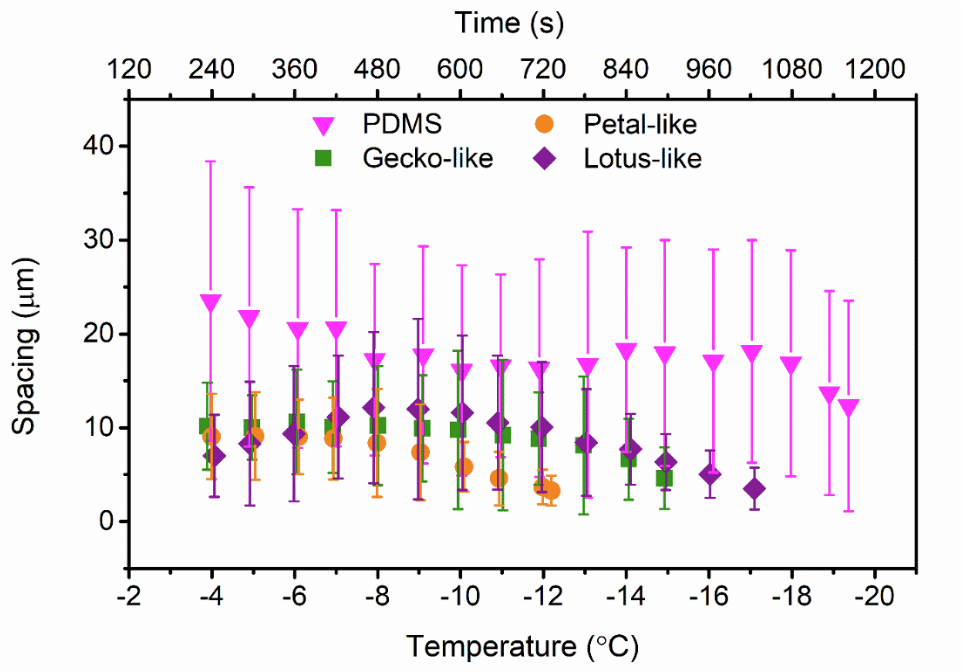

Figure S21. Condensate spacing test, related to Figure 7.
Plots of edge-to-edge spacing versus temperature/time for PDMS, gecko-like, petal-like, and lotus-like surfaces in condensation condition. Data are represented as mean ± SD.

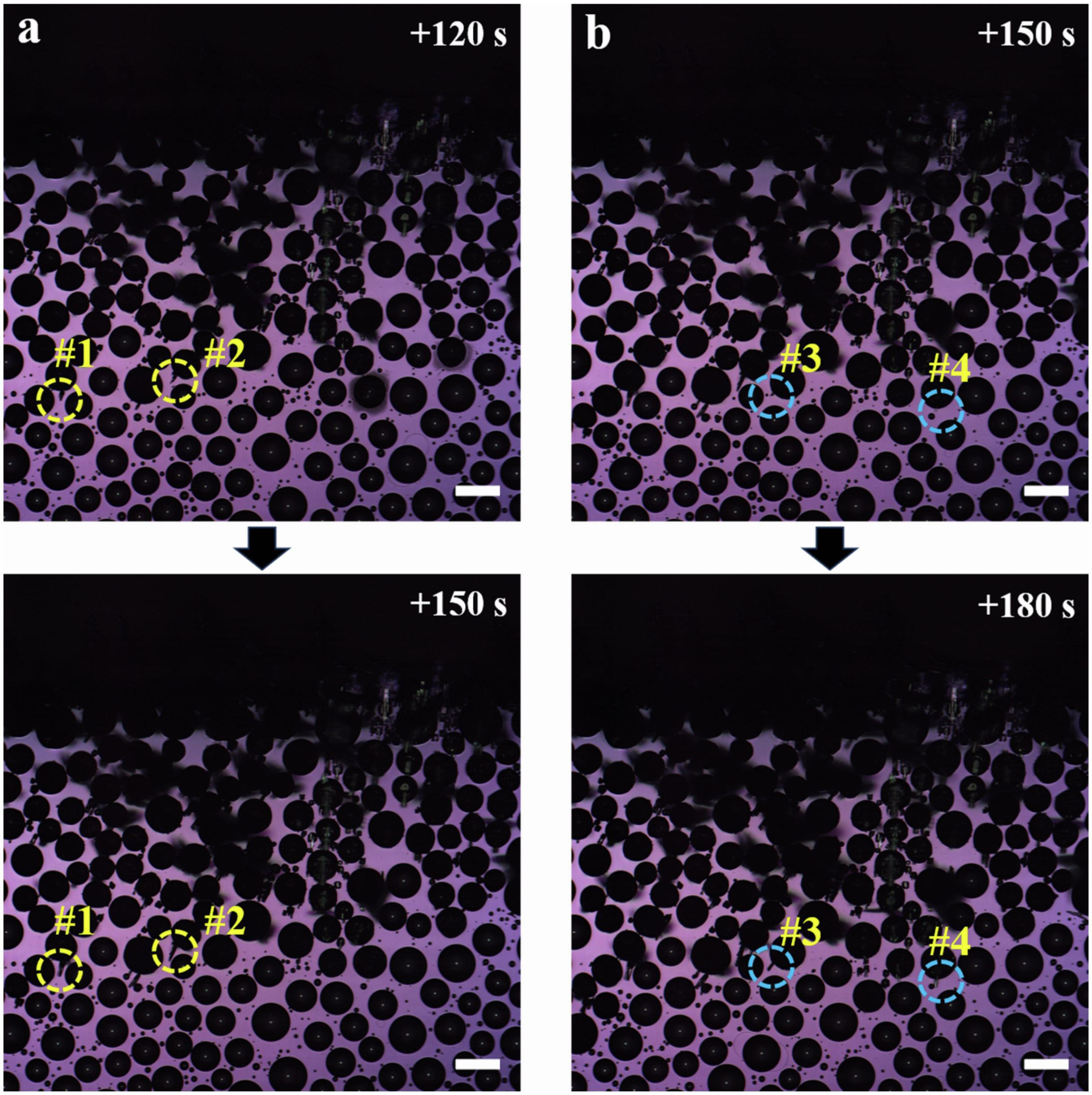

Figure S22. Ice bridge growth velocity test, related to Figure 7.
Time-lapse images for measuring the velocity of ice bridge growth on PDMS surface in condensation condition. Four ice bridges were circled. Scale bars are 250 μm.

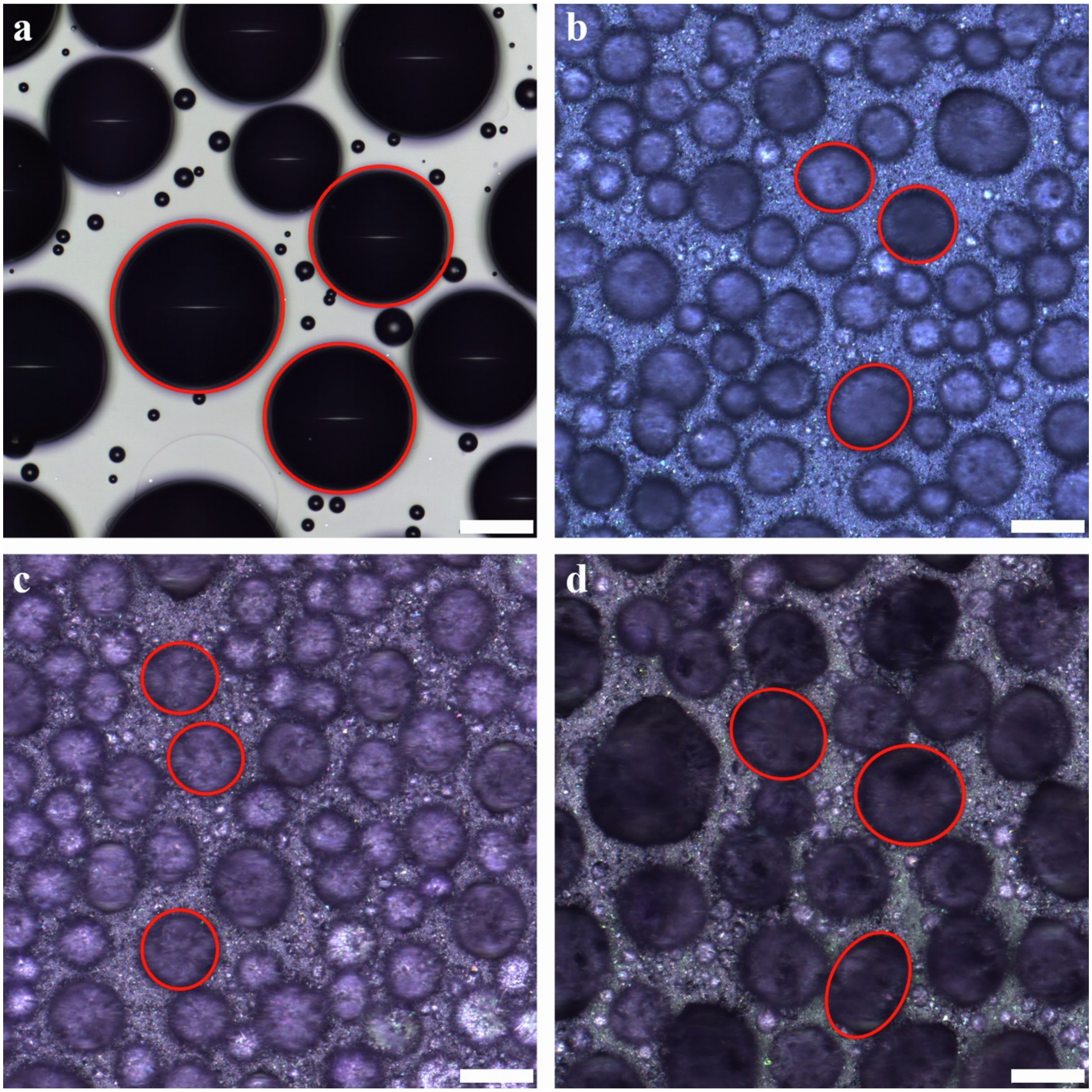

Figure S23. Targets for coalescence observation, related to Figure 8.
Three large condensed microdroplets upon freezing were chosen to check the coalescence process for (a) PDMS surface, (b) gecko-like surface, (c) petal-like surface, and (d) lotus-like surface. Scale bars are 100 μm.
